# EVITA Dengue: a cluster-randomized controlled trial to EValuate the efficacy of *Wolbachia*-InfecTed *Aedes aegypti* mosquitoes in reducing the incidence of Arboviral infection in Brazil

**DOI:** 10.1186/s13063-022-05997-4

**Published:** 2022-03-02

**Authors:** Matthew H. Collins, Gail E. Potter, Matt D. T. Hitchings, Ellie Butler, Michelle Wiles, Jessie K. Kennedy, Sofia B. Pinto, Adla B. M. Teixeira, Arnau Casanovas-Massana, Nadine G. Rouphael, Gregory A. Deye, Cameron P. Simmons, Luciano A. Moreira, Mauricio L. Nogueira, Derek A. T. Cummings, Albert I. Ko, Mauro M. Teixeira, Srilatha Edupuganti

**Affiliations:** 1 Department of Medicine, Division of Infectious Diseases, The Hope Clinic of the Emory Vaccine Center, Emory University, Atlanta, GA USA; 2 Biostatistics Research Branch, National Institute of Allergy and Infectious Diseases, National Institutes of Health, Rockville, MD USA; 3 The Emmes Company, LLC, Rockville, USA; 4 Emerging Pathogens Institute and Department of Biology, University of Florida, Gainesville, FL USA; 5 World Mosquito Program, Monash University, Melbourne, 3800 Australia; 6 School of Education, Universidade Federal de Minas Gerais (UFMG), Belo Horizonte, Minas Gerais Brazil; 7 Department of Epidemiology of Microbial Diseases, Yale School of Public Health, New Haven, CT USA; 8 Division of Microbiology and Infectious Diseases, National Institute of Allergy and Infectious Diseases, Bethesda, MD USA; 9 Instituto René Rachou, Fiocruz, Belo Horizonte, Minas Gerais Brazil; 10 Medical School of São Jose do Rio Preto FAMERP, São Jose do Rio Preto, São Paulo, Brazil; 11 Instituto Gonçalo Moniz, Fundação Oswaldo Cruz (Fiocruz), Salvador, Bahia, Brazil; 12 Department of Biochemistry and Immunology, Federal University of Minas Gerais, Belo Horizonte, Minas Gerais Brazil

**Keywords:** Arbovirus, Vector control, *Wolbachia*, Dengue, Zika, Chikungunya, Clinical trial, Prevention, Cluster-randomized controlled trial, Vector-borne disease

## Abstract

**Background:**

Arboviruses transmitted by *Aedes aegypti* including dengue, Zika, and chikungunya are a major global health problem, with over 2.5 billion at risk for dengue alone. There are no licensed antivirals for these infections, and safe and effective vaccines are not yet widely available. Thus, prevention of arbovirus transmission by vector modification is a novel approach being pursued by multiple researchers. However, the field needs high-quality evidence derived from randomized, controlled trials upon which to base the implementation and maintenance of vector control programs. Here, we report the EVITA Dengue trial design (DMID 17-0111), which assesses the efficacy in decreasing arbovirus transmission of an innovative approach developed by the World Mosquito Program for vector modification of *Aedes* mosquitoes by *Wolbachia pipientis*.

**Methods:**

DMID 17-0111 is a cluster-randomized trial in Belo Horizonte, Brazil, with clusters defined by primary school catchment areas. Clusters (*n* = 58) will be randomized 1:1 to intervention (release of *Wolbachia*-infected *Aedes aegypti* mosquitoes) vs. control (no release). Standard vector control activities (i.e., insecticides and education campaigns for reduction of mosquito breeding sites) will continue as per current practice in the municipality. Participants (*n* = 3480, 60 per cluster) are children aged 6–11 years enrolled in the cluster-defining school and living within the cluster boundaries who will undergo annual serologic surveillance for arboviral infection. The primary objective is to compare sero-incidence of arboviral infection between arms.

**Discussion:**

DMID 17-0111 aims to determine the efficacy of *Wolbachia*-infected mosquito releases in reducing human infections by arboviruses transmitted by *Aedes aegypti* and will complement the mounting evidence for this method from large-scale field releases and ongoing trials. The trial also represents a critical step towards robustness and rigor for how vector control methods are assessed, including the simultaneous measurement and correlation of entomologic and epidemiologic outcomes. Data from this trial will inform further the development of novel vector control methods.

**Trial registration:**

ClinicalTrials.govNCT04514107. Registered on 17 August 2020

Primary sponsor: National Institute of Health, National Institute of Allergy and Infectious Diseases

**Supplementary Information:**

The online version contains supplementary material available at 10.1186/s13063-022-05997-4.

## Background

Vector-borne diseases comprise some of the most notorious but also the most neglected diseases affecting global health [1]. Arthropod-borne viruses (arboviruses, ARBV) transmitted by the anthropophilic vector *Aedes aegypti* [2, 3] have been particularly prone to cause epidemics throughout the world [4, 5]. Over 2.5 billion people are at risk of infection by dengue virus (DENV) alone, and over 100 million dengue cases are reported each year [6–8]. In addition to dengue, Zika (ZIKV) and chikungunya viruses (CHIKV) are *Aedes*-borne viruses (ABV). One or more of the four DENV serotypes (DENV1-4) have become endemic in much of the tropical world. Meanwhile, the Americas have experienced large epidemics of CHIKV in 2013 [9] and ZIKV in 2015 [10] that rapidly spread through a large, susceptible population [11]. All three viruses commonly manifest as a fever and/or rash illness, though many cases are mild or asymptomatic such that they are not identified at health care facilities [12]. A small minority of dengue cases may be severe and even lead to death [13]. CHIKV infection is notable for its potential to cause “atypical chikungunya,” in which a severe illness is complicated by organ failure or arthritis that persists for months to years after infection [9, 14]. The ZIKV epidemic was declared an international public health emergency in 2016 due to its ability to cause congenital anomalies when transmitted from mother to fetus during pregnancy [15]. ZIKV infection has also been associated with neurologic disorders [16], sexual transmission [17, 18], and possible neurodevelopmental abnormalities following congenital or even post-natal infection [19–21]. Thus, interventions to control transmission and disease burden due to ABV are urgently needed. However, safe and effective vaccines or licensed antiviral medications for these infections are not yet available [5, 22, 23]. In this context, interventions targeting the mosquito vector to interrupt transmission of ABV could be an attractive component of a comprehensive strategy to control ARBV. However, the mounting case counts and ABV epidemics over the last decade begs the question as to which methods of control may be most effective.

A major issue that has hampered progress in this area is the lack of high-quality evidence upon which to base the implementation and maintenance of vector control programs [24, 25]. Until recently, the Camino Verde trial, which tested the impact of community mobilization on incident dengue infection and disease in a cluster-randomized controlled trial (CRCT), has been a unique example of systematic investigation of the efficacy of vector control interventions [26]. However, the field has been reinvigorated by the development of novel approaches to vector control that merit evaluation in well-designed field trials [1, 27, 28]. With the overall goal to reduce the burden of disease due to ABV, we designed a randomized controlled trial to test the efficacy of *Wolbachia*-infected (*w*Mel) mosquitoes in reducing transmission of DENV, CHIKV, and ZIKV. *Wolbachia*-infected mosquitoes have been shown to reduce the ability of the mosquito to transmit DENV [29, 30], ZIKV [31], and CHIKV [29, 32] as well as yellow fever [33] and Mayaro viruses in controlled laboratory experiments [34]. The *Wolbachia* method is safe, natural, and self-sustaining [30] and has the potential to achieve a significant public health impact in areas endemic to these viruses [35, 36]. In Townsville, Australia, early epidemiologic data shows that the establishment of *Wolbachia*-infected mosquitoes in the region (~140,000 inhabitants) eliminated local dengue transmission for more than four seasons, although imported dengue cases continued to occur [37]. In Indonesia, a CRCT in the city of Yogyakarta (400,000 inhabitants) began in 2018 using a case test-negative design (TN-CRCT), in which dengue cases and arbovirus-negative controls were sampled concurrently from febrile patients who presented to a network of primary care clinics in the intervention and control clusters. The strengths and weaknesses of this study design have been described [38], and this method was recently determined to reduce dengue cases by 77% in intervention areas [39]. Mathematical modeling suggests *Wolbachia* reduces the transmission potential of *A. aegypti* to the extent that elimination of DENV transmission could be possible in most settings [35]. In this setting, there remains a major need for robust evaluation of vector control methods in clinical trials assessing both entomologic and epidemiologic outcomes. More specifically, high-quality data for the *w*Mel-infected mosquito release method are essential to inform the full potential and implementation of this vector control strategy worldwide. In this article, we present the design and rationale for the EVITA Dengue trial, a school-based parallel arm cluster randomized control trial (PCRCT) in Belo Horizonte (BH), Brazil, designed to determine the effectiveness of *w*Mel mosquitoes in reducing the transmission of DENV, ZIKV, and CHIKV as indicated by annual incidence of infection by these ABV. Key challenges, solutions, and considerations involved in vector intervention trials are discussed. The trial is a partnership between Emory University Vaccine Trials Evaluation Network (VTEU) sponsored by the National Institute of Allergy and Infectious Disease (NIAID) Division of Infectious Diseases (DMID), the Brazilian Ministry of Health, the City of Belo Horizonte, and the World Mosquito Program.

## Methods/design

### Trial design

This is a two-arm parallel cluster-randomized trial with a target enrollment of 60 participants in each of 58 clusters (*n* = 3480). Participants are school children aged 6–11 years at enrollment who attend municipal schools within a participating school district in BH, Brazil. Participants will provide annual blood samples for serosurveys at baseline and annually for 3 years to assess for incident infections with DENV, ZIKV, and CHIKV. The trial intervention is the release of *Aedes aegypti* adult mosquitoes infected with *Wolbachia* (*w*Mel-BH). Clusters are randomized in a 1:1 ratio to the intervention or control (no release of *w*Mel-BH mosquitoes). All clusters will receive standard mosquito control as per current practice in the municipality of BH. The protocol is written consistent with the SPIRIT 2013 Statement, and a checklist of recommended protocol elements is provided in Additional file 1.

### Cluster definitions and characteristics

**Fig. 1:**
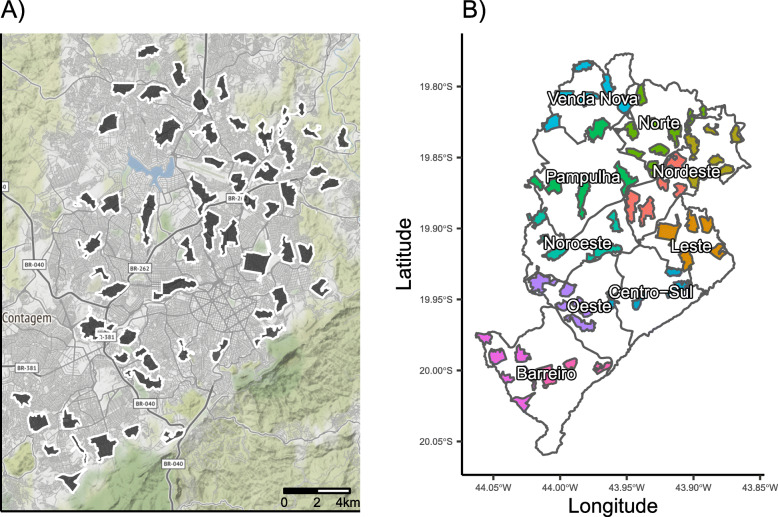
Spatial blocks used for randomization. **a** The political map of Belo Horizonte is shown with all enrolled clusters shown (*n* = 58). **b** The boundaries of the nine Distritos Sanitários (Health Districts) are shown in gray with color-coding indicating the spatial blocks used for randomization. Spatial blocks correspond to health districts except that the two districts with the largest number of clusters (Barreiro and Nordeste) were each divided into two spatial blocks. Overall allocation will be exactly 1:1 (intervention vs. control) and will be exactly 1:1 in blocks with even numbers of clusters and approximately 1:1 in blocks with odd numbers of clusters

Study clusters were designed based on specific primary school catchment areas, and a surrounding buffer zone separates all clusters from one another. To maximize the probability of introgression of the *Wolbachia*-infected mosquitoes and increase operational feasibility, the area of clusters was restricted to a size ≤2km^2^, with a minimum area target of 0.44km^2^. For catchment areas that were < 1km^2^, we redrew the boundary to define a cluster area of 1km^2^ that included the catchment area. For catchment areas that were > 2km^2^, we redrew the boundary to include the school and a contiguous populated area of 2km^2^ surrounding the school. Enrolled clusters are shown in Fig. 1a. To mitigate the risk of *Wolbachia*-infected mosquitoes invading control clusters, we chose to include clusters that were separated by a minimum distance of 200 m and/or by a natural boundary that inhibits mosquito movement. In BH, these natural boundaries were defined as *avenidas* and *rodovias*, which were generally roads of at least four lanes with a median barrier dividing the road into two lanes on either side.

### Study setting

**Fig. 2:**
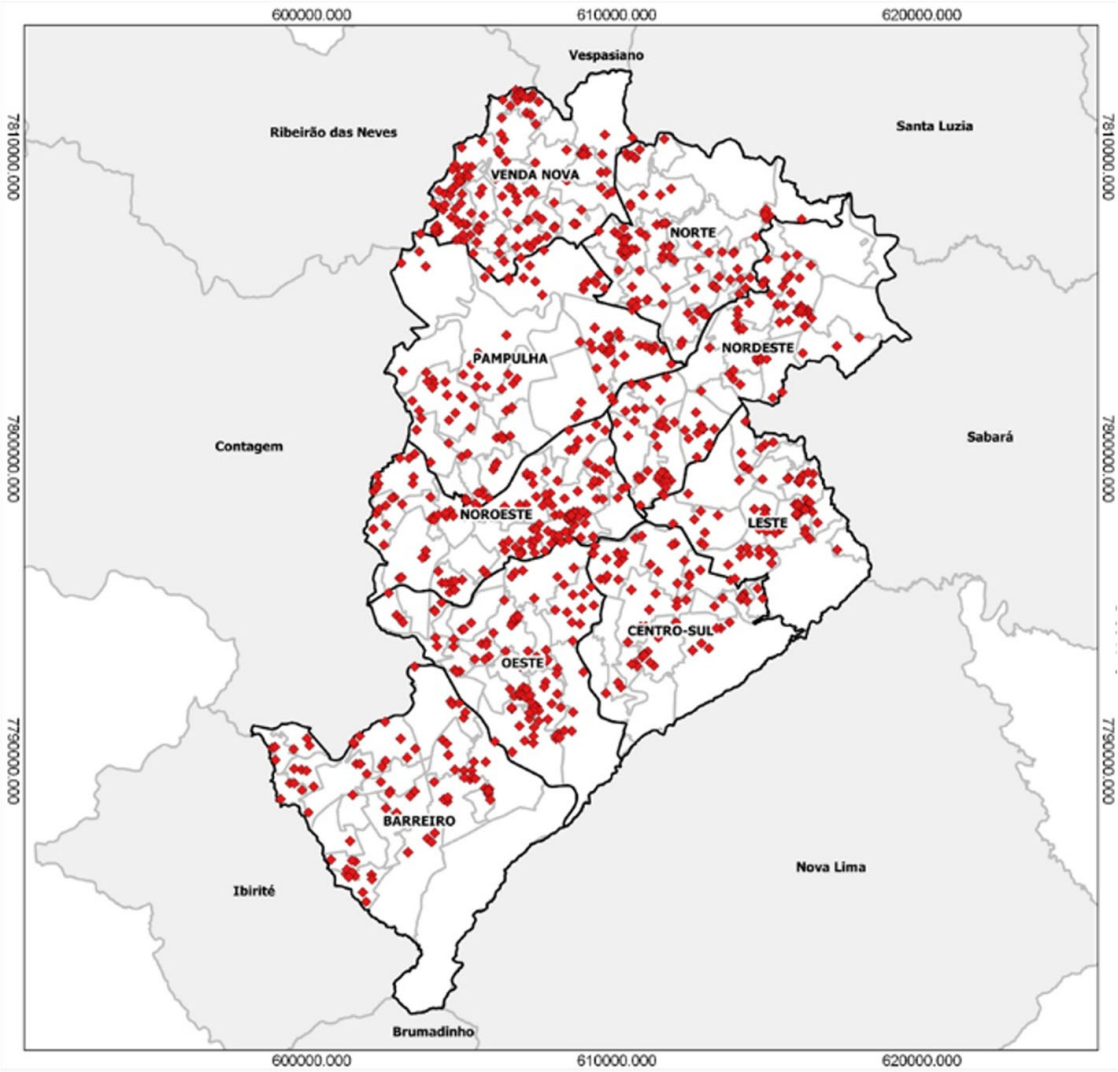
Confirmed cases of dengue in Belo Horizonte in 2017. Dark lines demarcate the nine health districts

**Fig. 3:**
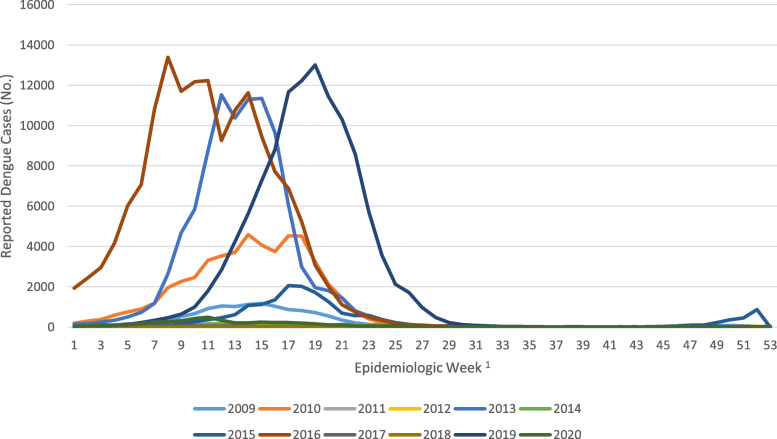
Dengue epidemiologic curves in Belo Horizonte, Brazil (2009–2020). ^1^Cases are recorded and displayed by the first day of symptom onset. Data depicted are obtained from publicly available data curated by the Health Department of the City of Belo Horizonte and available here: https://portalsinan.saude.gov.br

This trial is performed in BH, which is located in the state of Minas Gerais in southeastern Brazil and has a population of greater than 2.5 million. At 852 m above sea level and receiving approximately 50 inches of rain each year, the climate is tropical, with average temperatures ranging 17–20 °C [40]. There are several important reasons for selecting this site. First, dengue and other arboviral infections circulate in this region, with DENV epidemics registered in BH since 1996 [40]. Second, the epidemiology of BH is well characterized. The city is divided into 9 Distritos Sanitários (Health Districts), which keep a detailed record of confirmed DENV cases each year (Fig. 2). The annual epidemic curves for DENV over the last 9 years indicate typical peaks in March–May, but transmission may span from December to June, with large epidemics occurring every 3 or 4 years (Fig. 3). There is a dengue vaccine trial being conducted in a defined region in the western part of BH, and this region will not be included in this trial. Furthermore, BH will not qualify for introduction of Dengvaxia under new WHO guidelines [41]. Standard vector control programs are administered in the whole city and are in accordance with the Brazilian National Dengue Control Program (PNCD, Programa Nacional de Controle da Dengue) [42].

### Study population

Children aged 6–11 years old were enrolled from each of 58 participating elementary schools, each school defining a study cluster. For participation, one parent or legal guardian provided consent, and the child provided assent when age appropriate. Informed consent was administered by GCP-trained field study staff (“Agentes de campo”).

Inclusion criteria include that the child must be enrolled in the school that defines the cluster and the child must reside within the geographic boundaries of the cluster at least 5 days out of the week. Children will be excluded if there are plans to move to a new location outside the cluster boundary during the study period, the child has received a licensed or experimental vaccine against any ARBV of interest in this trial (DENV, ZIKV, CHIKV), the parent reports a medical condition that precludes safe donation of small blood volume, or the child has poor venous access. Dropout is defined in the protocol and includes participants who voluntarily withdraw, are lost to follow-up, or are withdrawn by the investigator because they no longer met eligibility criteria (e.g., moved out of their assigned cluster) or they are noncompliant. All blood samples will be processed at the Federal University in Minas Gerais (UFMG) in BH on the day of collection. Aliquots will be shipped to the central study lab in São José do Rio Preto for serologic analysis. The schedule of events is shown in Tables 4 and 5.

### Sample size

The sample size of 58 clusters with 60 children per cluster (*n* = 3480) was calculated to ensure at least 80% power (1-*β*) with a significance threshold (*α*) of 0.05 when comparing the incidence of arboviral infection between the intervention and control arms. The relationship between the number of clusters and the number of people per cluster was explored, and sixty people per cluster was selected as a feasible number to recruit, beyond which not much power is gained. Additional input parameters for the power calculation were based on existing literature and historical data from BH and are described below:

1. *Event rate of 13%:* To estimate the expected rate of seroconversion among schoolchildren, we estimated the annual hazard of dengue using reported age-specific case data in BH from 2016 to 2018, and from the state of Minas Gerais from 2008 to 2012, using the previously described method [43, 44]. We estimated annual per-serotype hazard of 0.025 (95% credible interval (CrI) 0.019, 0.027) in Minas Gerais from 2008 to 2012 and 0.029 (95% CrI 0.020, 0.033) for BH from 2016 to 2018 among children aged 6–8 years old at enrollment. Although we will target the 6–8-year age group for enrollment, we will allow children up to age 11 to enroll, and older children may have a slightly lower event rate. Therefore, we adjusted the assumed annual hazard to 0.022, and from this, estimated a cumulative incidence of dengue of 13% over 3 years of follow-up for children in our trial, assuming diagnostic sensitivity of 70% to detect true seroincident infection using annual serosurveys. This event rate accounts for the fact that a minor proportion of children will already have experienced multiple dengue infections at enrollment, and discerning additional DENV infections via annual serosurveillance may be difficult [45–47]. The selected event rate was based on the expected rate of DENV infections only, since there is not sufficient historical transmission data for ZIKV and CHIKV infections in BH. The rate of the primary outcome will be greater than or equal to that of dengue infections, so this approach is conservative.

2. *Effect size of 50%:* An effect size of 50% was selected as the minimum effect size of interest from a public health perspective while also being feasible in terms of the number of clusters needed to achieve adequate power.
3. *ICC of 0.07:* A value of 0.07 was selected for the intracluster correlation coefficient (ICC). In the Camino Verde trial, which has a similar endpoint and expected event rate to this trial, the ICC in the control group was 0.031 (95% *CI* [0, 0.11]) and it was 0.052 (95% *CI* [0, 0.16]) in the treatment group. The reported ICC from the Managua feasibility study was 0.18, but a confidence interval is not available [48]. These three estimates are based on 75, 75, and 20 clusters, respectively, so the 0.18 estimate is less precise. A value of 0.07 was selected as a reasonable value to power this trial while permitting a feasible sample size in terms of number of clusters. In calculating sample size, we assumed that the ICC will be the same in control and intervention arms. If the treatment effect is homogeneous (i.e., reduces all cluster-specific incidences by the same multiplicative factor), then the ICC will be lower in control than intervention clusters. We do not know whether our intervention effect will be homogeneous, but if it is, then power will be higher than planned, so our assumption is conservative.
4. *20% missingness (15% dropout and 5% under-enrollment):* The sample size calculation assumes that only 80% of participants will be analyzed, meaning that power is 80% if there is up to 20% missingness in the primary analysis. To allow for flexibility in meeting recruitment targets, the protocol allows the missingness to include up to 5% under-enrollment and up to 15% dropout. Power will be higher than 80% if missingness is less than planned. Furthermore, the protocol allows withdrawn participants to be replaced with age-matched children during annual serosurveys to minimize the impact of dropout on power.

Power was calculated with the clusterPower package in R version 3.4.2 [49] and validated with simulations using the planned analysis approach (Quasi-Poisson regression to compare incidence rates between arms). Both methods estimated 80% power for the given inputs. Power was also the same when an individual-level analysis approach with generalized estimating equations was tested in simulations.

### Intervention

The intervention is the release of *w*Mel-BH-infected *Aedes aegypti* adult mosquitoes. There are a few advantages of releasing adults rather than eggs as has been done previously [38]. Adult releases ensure that the actual intervention delivered reliably approximates the calculated number of adults entering the intervention area. The efficiency of introgression is also accelerated by the release of *w*Mel-BH-infected adults as these immediately begin to compete with WT adults, especially when *w*Mel males mate with WT females and cytoplasmic incompatibility precludes generation of WT progeny [50]. The strategy for the intervention in the EVITA Dengue trial in BH is informed by recent work by WMP. The WMP method was brought to Brazil in 2012 by Fundação Oswaldo Cruz (Fiocruz), based in Rio de Janeiro. Mosquito releases were done between 2014 and 2016 in pilot areas, Jurujuba, Niterói, and Tubiacanga, in the city of Rio de Janeiro. Since then, mosquito monitoring has revealed the successful introgression of *Wolbachia* with more than 90% of mosquitoes being *Wolbachia* infected years after mosquito releases ceased [51]. Based on WMP’s experience in the city of Belo Horizonte, the standard calculation for releases is 5.5 mosquitoes/person/week. Because clusters do not follow any census boundaries, a crude estimate of cluster population was made by using the average population density of the region multiplied by the area of the cluster. The nine health districts were stratified by conditions likely to predict a greater burden of DENV transmission such as population density, mosquito egg numbers in ovitraps, and total number of arboviral cases in the previous year. The value for each parameter is designated as high, medium, or low risk. The three risk parameters were designated as follows:

1. Population density: > 8000 inhab/km^2^ = high; between 8000 and 4000 inhab/km^2^ = medium; < 4000 inhab/km^2^ = low
2. Egg numbers/ovitrap between November and April from 2018 to 2020: > 75 eggs/trap = high; between 75 and 25 eggs/trap = medium; < 25 eggs/trap = low
3. Number of arbovirus cases in all of 2020: > 1000 cases/100,000 inhab = high; 1000 to 500 cases/100,000 inhab = medium; < 500 cases/100,000 inhab = low

High and low stratification factors change the number of mosquitoes needed by + 1 and −1 mosquito/inhabitant/week, respectively. For example, a region with high population density (+ 1) and high arbovirus cases (+ 1) would require 7.5 mosquitoes/inhabitant/week (5.5 + 1 + 1 = 7.5). Three regions were classified as high-risk and received 7.5 mosquitoes/person/week, and the rest received 5.5 mosquitoes/person/week.

The deployment of *Wolbachia*-infected *A. aegypti* mosquitoes consists of two phases: (1) a 16-week establishment phase in which most of the releases will occur and (2) a 16-week consolidation phase in which the prevalence of *Wolbachia* among *A. aegypti* mosquitoes is measured weekly and remedial deployments are completed if an intervention cluster does not reach 60% prevalence of *Wolbachia*-infected *A. aegypti* or if it drops below 60% [50].

Independent of trial activities, both control and intervention clusters continue to receive standard vector control as administered by the national dengue control program in Brazil (PNCD, Programa Nacional de Controle da Dengue). This program has 4 basic principles: (i) adequate case finding, classification, and treatment; (ii) epidemiological surveillance and report of all cases; (iii) mobilization and communication of risks to the public; (iv) mosquito monitoring and control which consists fundamentally of detection of larva using a rapid larval index (LIRAa by its initials in Portuguese) followed by removal of breeding sites and local spraying. The LIRAa is performed four times per year and guides control efforts in the area. In addition to the recommendation by the PNCD, the city of Belo Horizonte conducts a detailed larval survey of the entire city with ovitraps.

### Randomization

The randomization of clusters incorporated spatial blocking and covariate constraints to ensure that the two intervention arms are balanced with respect to key predictors of arboviral infection. The study area was divided into eleven spatial blocks corresponding to the nine health districts in the city, with the two largest of the nine districts each being divided into two spatial blocks. Health districts were selected for blocking, as they tend to have distinct geographic and socioeconomic profiles, so the risk of arboviral infection is expected to be similar within spatial blocks but potentially different between blocks. Figure 2b shows the blocks for the 58 planned clusters. Schools were prioritized for recruitment based on the number of 6–11-year-old children at the school, the size of the catchment area, and whether sufficient space separates it from all other candidate schools. As per the protocol, schools for a given spatial block were randomized only after all subjects have been enrolled for all schools within that block. This approach was intended to prevent potential bias that could arise from differential enrollment between treated and control clusters if some participants are able to decipher their treatment assignments. For blocks with even numbers of clusters, randomization was 1:1 within the block. For those with odd numbers of clusters, the number of treated and control assignments within the block differed by at most 1, and the larger number of assignments will be assigned to either category (intervention or control) with equal probability. The randomization procedure ensured 29 clusters in each arm. In addition to spatial blocking, certain covariate constraints were imposed to prevent chance imbalance between intervention and control arms at baseline (Table 1). Covariates were constrained to ensure balance between arms, but not necessarily within blocks. The randomization assignments were generated through simulation in R statistical software [52] by a statistician at Emmes, the statistical and data coordinating center for this trial, as follows:

- One million allocations satisfying the spatial blocking criteria and with exactly 29 clusters per arm were simulated (set A).
- All allocations satisfying the covariate constraints described above were selected from set A (set B).
- One of the allocations from set B was selected at random.

**Table 1:** Covariates for constrained randomization

Covariate	Rationale	Balancing criterion
Socioeconomic status (SES), measured by dichotomizing INSE scores into two categories (> 3 vs ≤3)	SES may predict arboviral infection risk	Each arm within ±5% of overall proportion
Population density	Population density may predict arboviral infection risk	Each arm within ±5% of overall population density
Number of students in grades 1 and 2 combined	Proxy for age of participants to be enrolled in a given cluster. Age is predictive of individual arboviral infection risk.	Each arm 45–55% of total number

The allocation list was electronically transmitted as two separate files corresponding to the two groups of spatial blocks that enrolled in a staggered fashion. Each file was sent by providing a secure website link only viewable via the password-protected account of WMP’s Head of Operations for this study. After download, the list was stored in a secure location and removed from the secure area of the website.

### Blinding and concealment

School staff and participants and their families will not be informed of their treatment arm, and surveillance to collect mosquitoes during the deployment phase in control clusters will be identical to intervention clusters. These procedures should reduce the probability that participants become aware of their intervention arm status. However, mock releases will not be performed in control clusters. To evaluate how effective our blinding was, we will conduct annual surveys of all participants’ parent/legal guardian to assess their knowledge of their study arm assignment. Principal investigators will not have access to intervention arm assignment nor will study staff engaged in monitoring of mosquito populations, laboratory staff, or study staff interacting with participants. Study staff who deliver *Wolbachia*-infected mosquitoes in the intervention arm will, by necessity, know the treatment assignment. However, this team will be separate staff than staff performing mosquito monitoring and other aspects of the trial. The mosquito releases themselves cannot be completely concealed from community members, primarily due to local regulatory and safety concerns that stipulate that vehicles be labeled, and staff be in uniform. However, most releases are accomplished in early morning hours when fewer residents are outside of their homes. Cluster allocations are concealed from all study staff, including investigators and those involved with mosquito production, trap monitoring, *Wolbachia* testing, and community engagement, except for approximately 10 essential personnel managing deployment.

### Objectives and endpoints

The primary and secondary objectives, endpoints, and outcomes measures are summarized in Table 2. These are generally divided into epidemiologic and entomologic outcomes, consistent with leading recommendations that concurrent data collection in these two domains is essential for the highest quality trial evidence and a comprehensive understanding of how to control dengue and other ARBV. The primary objective is to evaluate whether the release of *Wolbachia*-infected *Aedes aegypti* mosquitoes *plus* standard *Aedes* vector control measures reduce the sero-incidence of ARBV (DENV, ZIKV, CHIKV) infection compared to standard *Aedes* vector control measures alone.

**Table 2:** Objectives, outcome measures, and endpoints

**Primary objective, outcome measure, and endpoint**		
**Objective**	**Outcome measure**	**Endpoint**
1. To evaluate whether the release of *Wolbachia*-infected *Aedes aegypti* mosquitoes *plus* standard *Aedes* vector control measures reduces the sero-incidence of ARBV infection compared to standard *Aedes* vector control measures alone.	Incident ARBV infection is defined as seroconversion to DENV, ZIKV, or CHIKV, as detected during annual serological evaluations.	Seroconversion, defined as an initial negative titer (< 1:20) and subsequent titer ≥1:20 in FRNT50 testing of sequential annual samples OR ≥ fourfold increase in titer in FRNT50 testing of sequential annual samples with one or more FLAV (DENV1, DENV2, DENV3, DENV4, or ZIKV). Seroconversion for CHIKV, defined as IgG ELISA initial conversion from negative to positive. Seronegative is defined as FRNT50 < 1:20 for FLAV and IgG ELISA negative for CHIKV.
**Secondary objectives, outcome measures, and endpoints**		
**Objective**	**Outcome measure**	**Endpoint**
1. To evaluate whether the release of *Wolbachia*-infected *Aedes aegypti* mosquitoes plus standard *Aedes* vector control measures reduces the sero-incidence rate of ARBV infection, inferred from a model-based reconstruction of serological dynamics compared to standard *Aedes* vector control measures alone.	ARBV infections, specifically due to FLAV or CHIKV, as detected during annual serological evaluations, inferred from a model-based reconstruction of serological dynamics.	Model estimated infection based on the reconstruction of serological dynamics.
2. To evaluate whether the release of *Wolbachia*-infected *Aedes aegypti* mosquitoes *plus* standard *Aedes* vector control measures reduces the sero-incidence of FLAV or CHIKV infection among individuals who are seronegative to each of these families of viruses, respectively, at study entry, compared to standard *Aedes* vector control measures alone.	Sero-incidence of FLAV or CHIKV infection as detected during annual serological evaluations in the sub-group of participants who are seronegative to each of these families of viruses, respectively.	Seroconversion will be measured for the subgroup of participants who are ARBV seronegative (FRNT50 < 1:20 for FLAV or IgG ELISA negative for CHIKV) at study entry.
3. To evaluate whether the release of *Wolbachia*-infected *Aedes aegypti* mosquitoes plus standard *Aedes* vector control measures reduces the overall sero-incidence of FLAV (DENV + ZIKV) infection.	FLAV infection as detected during annual serological evaluations.	Seroconversion, defined as an initial negative titer (< 1:20) and subsequent titer ≥1:20 in FRNT50 testing of sequential annual samples OR ≥ fourfold increase in FRNT50 titer of sequential annual samples with one or more FLAV (DENV1, DENV2, DENV3, DENV4, and ZIKV).
4. To evaluate whether the release of *Wolbachia*-infected *Aedes aegypti* mosquitoes plus standard *Aedes* vector control measures reduces the sero-incidence of DENV infection.	DENV infection as detected during annual serological evaluations.	Seroconversion, defined as an initial negative titer (< 1:20) and subsequent titer ≥1:20 in FRNT50 testing of sequential annual samples OR ≥ fourfold increase in FRNT50 titer of sequential annual samples with one or more DENV serotypes; AND ZIKV FRNT50 titer does NOT increase ≥ fourfold.
5. To evaluate whether the release of *Wolbachia*-infected *Aedes aegypti* mosquitoes plus standard *Aedes* vector control measures reduces the sero-incidence of ZIKV infection.	ZIKV infection as detected during annual serological evaluations.	Seroconversion, defined as an initial negative titer (< 1:20) and subsequent titer ≥1:20 in FRNT50 testing of sequential annual samples OR ≥ fourfold increase in FRNT50 titer of sequential annual samples with ZIKV; AND FRNT50 titer does NOT increase ≥ fourfold for any DENV serotype.
6. To evaluate whether the release of *Wolbachia*-infected *Aedes aegypti* mosquitoes plus standard *Aedes* vector control measures reduces the sero-incidence of CHIKV infection.	CHIKV infection as detected during annual serological evaluations.	Seroconversion for CHIKV, defined as IgG ELISA initial conversion from negative to positive.
7. To evaluate whether the release of *Wolbachia*-infected *Aedes aegypti* mosquitoes *plus* standard *Aedes* vector control measures reduces the sero-incidence of infection with a DENV serotype among individuals who are seropositive to another DENV serotype(s) at study entry, compared to standard *Aedes* vector control measures alone.	Infection with a DENV serotype as detected during annual serological evaluations of the sub-group of participants who are seropositive to another DENV serotype(s).	Infection with a new DENV serotype for the subgroup of participants who are seropositive (≥1:20) to a different DENV serotype(s) at study entry.
8. To evaluate the extent to which *Wolbachia*-infected *Aedes aegypti* mosquitoes replace uninfected adult *Aedes aegypti* in intervention clusters.	Proportion of *Wolbachia*-infected *Aedes aegypti* adults in intervention clusters at specified time points	Proportion of *Wolbachia*-infected (PCR-positive) *Aedes aegypti* adults in intervention clusters.
9. To evaluate the contamination of control clusters by *Wolbachia*-infected *Aedes aegypti* mosquitoes released in intervention clusters.	Proportion of *Wolbachia*-infected *Aedes aegypti* adults in control clusters at specified time points.	Proportion of *Wolbachia*-infected (PCR-positive) *Aedes aegypti* adults in control clusters.

Assessment of the primary outcome (and several secondary outcomes) is accomplished through serologic testing [53]. Definitions of endpoints are included in Table 2. The outcome of interest is incident ABV infection, which is satisfied by serologic evidence of incident infection by CHIKV or a flavivirus (ZIKV or any serotype of DENV) as detected by annual serosurveillance. CHIKV infection is determined by IgG ELISA. Flavivirus infection is detected by testing for neutralizing antibodies in a focus reduction neutralization test (FRNT). In subjects with no prior flavivirus history, seroconversion to one or more flaviviruses is equal to one event for the study year being examined. Extensive neutralization testing will be performed with the goal of distinguishing the virus/serotype causing the incident infection. Detection of a fourfold rise in neutralizing antibody titer is required to identify an incident flavivirus infection among children with evidence of previous flavivirus infection at the beginning of the study period being analyzed.

### Statistical analysis plan

The primary objective of this study is to compare ARBV incidence among 6- to 11-year-olds between intervention and control arms. A cluster-level analysis is planned, in which the observations are the total numbers of ARBV infections per cluster and participants may experience multiple infections during follow-up. Quasi-Poisson regression will be performed including person-years of follow-up per cluster as an offset, in order to estimate the incidence rate ratio (IRR) between treatment arms. Quasi-Poisson regression is a flexible model for count distributions which allows for potential overdispersion, is robust to model misspecification, and is an appropriate model for our design [54]. A supporting analysis will adjust for covariates, which may include the geographic size of cluster, baseline seropositivity, socioeconomic status, and baseline mosquito prevalence. The primary analysis will be on the intention-to-treat population, including participants according to their randomization assignment and censoring them at the time of dropout or time of moving out of their cluster.

A secondary per-protocol analysis was planned to include clusters that have achieved a ≥ 60% proportion of *Wolbachia*-infected mosquitoes among the trapped *A. aegypti* (done during May and June of the first dengue season after deployment). Because of delays in deployment due to the COVID-19 pandemic, this definition was modified to exclude time-at-risk and events during the first year of the trial. See further discussion of this in the section “Trial adaptations to challenges posed by the COVID-19 pandemic” below. The modified per-protocol analysis definition includes intervention arm clusters that have achieved a ≥ 60% proportion of *Wolbachia*-infected mosquitoes among the trapped *A. aegypti* expressed as an average of the mosquito surveillance collections done during the consolidation phase. The per-protocol analysis will include all control arm clusters in a randomization block where at least one intervention arm cluster fulfills the per-protocol criterion. Subjects will be included in the per-protocol population (1) if they reside in either the assigned cluster or another cluster with the same per-protocol treatment status throughout the arboviral transmission seasons of Feb–Jun 2022 and/or Feb-Jun 2023 and (2) if they provided a paired sample in 2021 and 2022 and/or a paired sample in 2022 and 2023. Person time and outcomes between 2021 and 2022 and 2022 and 2023 blood collection surveys will be included in the per-protocol analysis while person time and outcomes between 2020 and 2021 surveys will be excluded from the analysis.

The study includes two adaptive analysis strategies allowing a flexible follow-up period (1 to 4 years). Historical data show that years with high dengue incidence are often followed by dry spells. In BH, the decade from 2008 to 2017 shows that 3 years of high incidence (2010, 2013, 2016) were each followed by 2 years of very low incidence (Fig. 3) [55]. This temporal variability of dengue could cause power to be lower than planned if dengue is rare during the planned 3-year follow-up period or higher than planned if an epidemic is observed early in the study. The adaptive approaches allow the follow-up period to adapt in response to such scenarios in order to keep actual power close to the planned power. First, to safeguard against an event rate lower than the planned 13% over the 3-year follow-up period, the aggregate seroconversion rate will be calculated before conducting the final analysis at the end of the follow-up. If the intervention has a 50% effect size as assumed, then the 13% event rate in the control group needed for at least 80% power would mean the event rate in the treatment group should be around 6.5%, corresponding to an aggregate event rate of at least 9.75%. The trial may be extended, contingent upon available funding and sponsorship, for an additional year of follow-up if the overall event rate is < 9.75% at the end of the 3-year follow-up. Second, it is possible that the event rate of dengue seroconversions in the control group will be larger than the planned 13%, and in fact, the planned event rate may be exceeded even in 1 year of the study alone. The aggregate (blinded) seroconversion rate will be calculated twice: at the end of year 1 and the end of year 2. If the aggregate seroconversion rate is at least 34%, the trial will be stopped early, and the final analysis performed. The threshold of 34% was selected by calculating power for various aggregate event rates and effect sizes (Table 3). This threshold ensures at least 90% power to detect an effect size as low as 30%. Both of these adaptive strategies are based on blinded analyses, so alpha-spending adjustments are not needed to preserve the type 1 error rate [56]. The two adaptive aspects mean that, although a 3-year follow-up period is planned (Tables 4 and 5), the total follow-up period can range between 1 and 4 years.

**Table 3:** Power available for various aggregate seroconversion rates and effect sizes. Note: The interim seroconversion rate in the control arm was derived algebraically from the observed aggregate rate and the effect size

Interim aggregate seroconversion rate (threshold for stopping)	50% effect size		40% effect size		30% effect size	
	Interim seroconversion rate in control arm	Power	Interim seroconversion rate in control arm	Power	Interim seroconversion rate in control arm	Power
10%	13%	81%	12%	56%	12%	32%
15%	20%	95%	19%	76%	18%	47%
20%	27%	99%	25%	89%	24%	61%
25%	33%	> 99%	31%	96%	29%	74%
30%	40%	> 99%	37%	99%	35%	84%
33%	44%	> 99%	41%	99%	39%	88%
34%	45%	> 99%	42%	99%	40%	90%
35%	47%	> 99%	44%	> 99%	41%	91%
40%	53%	> 99%	50%	> 99%	47%	96%

**Table 4:** Schedule of individual assessments

Evaluation	Screening Visit 00	Enrollment Visit 01 Year 1	Parental contact Visit 01P^**d**^ Year 2	Visit 02^**a**^ Year 2	Time Use Survey^**b**^	Parental contact Visit 02P^**d**^ Year 3	Visit 03^**a**^ Year 3	Time Use Survey^**b**^	Parental contact Visit 03P^**d**^ Year 4	Final visit Visit 04^**a**^ Year 4
Study timepoint	−60 to 0 prior to visit 01	Sep–Dec 2020	−60 to 0 day(s) prior to visit 02	Jun–Nov 2021	Feb–Jun 2022	−60 to 0 day(s) prior to visit 03	Jun–Nov 2022	Feb–Jun 2023	−60 to 0 day(s) prior to visit 04	Jun–Nov 2023
Signed consent form^c^	X									
Minor assent form	X									
Eligibility review	X		X			X			X	
Demographic survey^c^	X									
Collection of relevant medical conditions^c^	X									
Assessment of eligibility	X	X								
Phlebotomy		X		X			X			X
Parental contact^d^				X			X			X
Knowledge of treatment arm survey^c, e^				X			X			X
Time Use Survey					X			X		

^a^Visit will occur after the peak transmission season (Feb–June)

^b^Visit will only be completed by a subset of participants enrolled in the Time Use Survey Sub-Study

^c^To be completed by the participant’s parent/legal guardian −60 to 0 days prior to the blood draw

^d^Includes confirmation of continuing study participation and collection of any significant health updates that would make the participant ineligible for study activities

^e^To be completed during parental contact

**Table 5:** Schedule of entomological assessments during establishment and consolidation phase (Jan–Dec 2021)

	Jan	Feb	Mar	Apr	May	Jun	Jul	Aug	Sep	Oct	Nov	Dec
**Low season**	**x**						**x**	**x**	**x**	**x**	**x**	**x**
**High season**		**x**	**x**	**x**	**x**	**x**						
**Procedure**												
*Wolbachia*-infected nosquito release	x^a^	x^a^	x^a^	x^a^	x^a^	x^a^	x^a^	x^a^	x^a^	x^a^	x^a^	x^a^
Adult population monitoring		w	w	w	w	w	w	w	w	w	w	w
PCR testing		bw	bw	bw	bw	bw	bw	bw	bw	bw	bw	bw

*m* monthly, *w* weekly, *bw* biweekly (every 2 weeks)

^a^Initial release in all clusters to occur over a minimum of 16-week period

### Study implementation

The development of the study protocol, as well as other documents and tools such as case report forms (CRFs) and the Manual of Procedures (MOP), followed standard guidance and approval processes established by the US National Institutes of Health, specifically the Division of Microbiology and Infectious Diseases (DMID). Data entry and the study database is managed by a contracting clinical research organization, which has several built-in quality checks. The study is formally audited quarterly by a contracting organization selected by DMID. Prior to the start of the enrollment of study participants, community engagement events and media releases were implemented. Study leadership also initiated engagement with the Vector Control Advisory Group (VCAG) of the World Health Organization.

### Trial adaptations to challenges posed by the COVID-19 pandemic

**Fig. 4:**
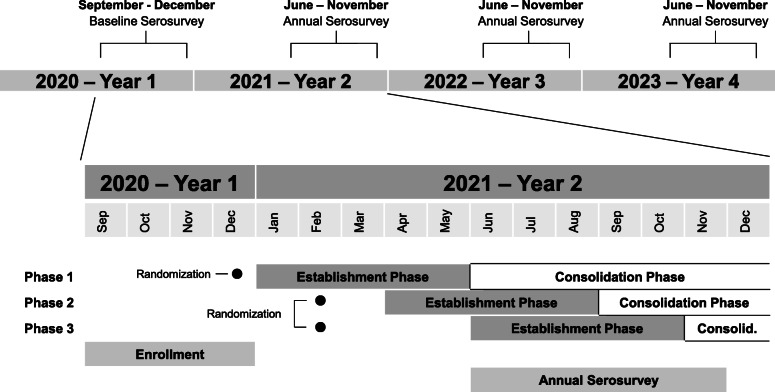
EVITA Dengue study timeline. The top line displays the 4 years of the trial, indicating when serosurveillance samples were obtained from participants, which occurred in the low transmission season each year. The lower portion is an expanded view of year 1 and year 2 to better show the timing of intervention deployment in relationship to other study activities. Randomization 1 occurred on December 9, 2020. Randomization 2 occurred on February 22, 2021

This trial was implemented under extraordinary circumstances, namely the COVID-19 pandemic, which required certain adaptive allowances in the design. Enrollment at schools was originally expected to start in mid-2020. However, due to COVID-19, schools were closed in 2020, thus requiring home-based recruitment and enrollment. Despite these challenges, target enrollment was achieved in 2020. Delivery of the intervention was planned to be completed prior to the earliest time in the transmission season (January) in 2021 (Additional file 2). However, supply chain and mosquito production capacity were interrupted due to COVID-19. As such, the mosquito intervention was implemented in three groups of spatial blocks that were staggered in time (Fig. 4). Because randomization was stratified on the spatial block (see the “Randomization” section above), a staggered approach will not introduce bias that could arise from differential enrollment time between treatment arms. In addition, because deployments occurred during the cold season, which has low mosquito prevalence, the measurements of *w*Mel introgression in intervention clusters were based on small numbers of trapped mosquitoes, making it difficult to confirm if 60% prevalence had been achieved. Therefore, the consolidation phase of the intervention was extended to an 8-month duration. The delay in delivery of intervention means that clusters experienced a watered-down treatment effect during the first year of observation (2020–2021). This important risk to the trial is mitigated by three key factors. First, dengue epidemics tend to occur once every 3 years in the study area, with high years typically separated by two low years (Fig. 3), and according to publicly available information on dengue cases in the region of BH, 2021 appears to have been a very low year for case numbers. Therefore, most events that define the primary endpoints (and many secondary endpoints) are likely to occur in the second or third year of the trial. The incidence rate ratio analysis is driven by time intervals during which events are observed, so the inclusion of an interval with a watered-down treatment effect will have a minimal statistical impact on the primary analysis if few or no events were observed during that interval. Second, to address the unlikely possibility that substantial asymptomatic transmission occurred in 2020–2021, the per-protocol (PP) population definition was revised to exclude time-at-risk and events from 2020 to 2021 and analyze only the second 2 years of the trial. With this approach, clusters should experience a full treatment effect during time intervals included in the PP analysis. Finally, the study protocol includes the option for extending the observation period by 1 year if the event rate is lower than expected over the three observation periods planned.

### Ethical considerations

The Brazilian and US regulatory requirements (governmental and institutional) were fully adhered to in the planning of this trial. Informed assent will be obtained for the pediatric population in an age-appropriate manner in addition to parental consent. From a human subject perspective, the study is considered minimal risk since phlebotomy is the only study procedure. In terms of confidentiality, participation in the study is likely to be known by peers at the school and perhaps in the community, which is culturally and ethically acceptable for this subject matter. The resolution of any images containing geocoding data will not allow the identification of individual households. The study was reviewed and approved by the Federal University of Minas Gerais Research Ethics Committee and the Emory University Institutional Review Board. In addition to institutional approvals, approval of the Brazilian National Human Ethics review board (CONEP) has been obtained. A data and safety monitoring board (DSMB) composed of independent Brazilian and American scientists and public health officials has been assembled and meets annually.

There are unique and interesting ethical considerations for a trial that involves a biologic intervention performed on the environment rather than human research subjects (distinct from classic intervention trials with vaccines, drugs, or medical devices). A full discussion of these considerations is beyond the scope of this trial protocol [57]. A key feature is that individual informed consent is obtained from participants who are volunteering to donate specimens for annual serosurveillance. The informed consent decision is whether to donate biological specimens and be monitored by the study team, not whether to receive the intervention. People living in the study area may receive the mosquito intervention in their environment without a specific individualized consent process. Rather, deployment of the intervention is approved by local and federal governance, which represents the interest of its constituents living in the study area. Brazilian authorities have deployed *Wolbachia*-infected mosquitoes in the municipalities of Rio de Janeiro since 2014. Expansion of *w*Mel mosquito deployments to the municipality of BH was planned for 2020 irrespective of the EVITA Dengue trial. The EVITA trial has been able to synergize with the *w*Mel mosquito deployments in BH and is poised to generate RCT-level data evaluating the efficacy of this vector control intervention.

The trial is funded by the US National Institutes of Health (Division of Microbiology and Infectious Diseases), the City of Belo Horizonte, and Brazilian Ministry of Health. As per the US-based regulatory requirements (Guidance for Industry #236 – Clarification of Food and Drug Administration (FDA) and Environmental Protection Agency (EPA) Jurisdiction over Mosquito-Related Products) for mosquito-related products intended to cure, mitigate, treat, or prevent a disease (including by an intent to reduce the level, replication, or transmissibility of a pathogen in mosquitoes), are considered “drugs” under the Federal Food, Drug, & Cosmetic Act. However, the *Wolbachia*-infected mosquitoes will be neither manufactured nor deployed in the USA. Therefore, FDA oversight is not required in this trial.

In Brazil, the use of *Wolbachia*-infected *A. aegypti* was granted regulatory and ethical approvals in 2014. The Temporary Special Registry (Registro Especial Temporário - RET, in Portuguese) was granted in 2014 after evaluating the project simultaneously by three governmental areas: National Agency of Sanitary Surveillance - ANVISA; Ministry of Agriculture, Livestock and Supply (MAPA) and the Brazilian Institute of Environment and Renewable Natural Resources (IBAMA). Ethical approval was also granted on March 5, 2014, following an evaluation by the National Commission for Research Ethics (CONEP). For the large-scale expansion project, the RET and ethical approval were renewed in 2017 and 2016, respectively.

### Community and stakeholder engagement

Community and stakeholder engagement (CSE) is long recognized as critical to public health efforts such as those described here, and it is increasingly appreciated that systematic approaches and standardized frameworks may broadly benefit human health and development activities and implementation of novel vector control strategies specifically [58–60]. An extensive discussion of this topic is beyond the scope of this manuscript, but key elements included in the study protocol are summarized briefly. CSE activities were organized around (1) engagement with school communities (children, families, school personnel, and education administration officials) surrounding the establishment of a pediatric surveillance cohort and (2) engagement with the general community surrounding the implementation of *Wolbachia*-infected mosquito releases for ARBV transmission control.

1. Local school governance, including the city’s Secretary of Education, has been involved. Approval was obtained at the school level and individual points-of-contact identified among the teachers at each school. For participating schools, a “Dengue Day” health fair event will be arranged to further engage, inform, and foster dialogue with each school community.
2. WMP has included CSE as a key component of its framework since its founding in 2011 and has recent and relevant experience in Brazil [59, 61–63]. There is already strong support and enthusiasm for WMP activities from local officials in the health ministry. Community advisory boards will be established to maintain channels of communication. Local television and radio, and social media will also be used for messaging.

## Discussion

There remains a critical need to improve the evidence base for guiding vector control practice and policy, particularly for ABV, and it is equally important to systematically assess the efficacy of traditional as well as novel approaches to vector control [1, 25]. We therefore designed a PCRCT to evaluate the efficacy of releasing *Wolbachia*-infected mosquitoes in reducing ARBV infection, which is considered a gold standard to determine the efficacy in vector-control trials [54, 64, 65]. The CRCT provides a robust design to evaluate interventions administered at the community level rather than individual level [66]. Alternative design choices include the stepped wedge design and cluster-randomized test-negative design. In a stepped wedge design, all clusters experience both control and intervention conditions and cross over from control to intervention at randomly assigned time points. The treatment effect estimate is informed by both within-cluster and between-cluster differences in outcomes between control and intervention conditions [67]. Simulations have demonstrated that this design is less powerful than a CRCT for studying dengue incidence due to the spatial and temporal heterogeneity of dengue, and this design cannot flexibly incorporate adaptive follow-up periods [54]. The cluster-randomized test-negative design (CR-TND) is similar to a CRCT in randomizing clusters to intervention versus control, but differs in its sampling strategy of participants. A CR-TND is a type of case-cohort design in which individuals presenting in health clinics with symptoms are enrolled; those who test positive for the infection of interest are cases and those who test negative are controls. For this design to be valid, several conditions must be met [68]. One condition is that test-negative illness is not associated with the intervention—a condition which would be violated if the release of *Wolbachia*-infected *Aedes* mosquitoes reduces cases due to etiologies other than the ARBV for which specific testing is performed. Another condition is that intervention effectiveness is unrelated to health-care–seeking behavior. This could be violated if healthcare-seeking behavior is related to socioeconomic status (SES), and if the intervention is variable in low SES areas versus high SES areas. These conditions are not a threat to the CRCT design described here.

For valid causal inference using traditional CRCT analysis methods, participants’ outcomes must not be influenced by treatment conditions outside of participant’s assigned cluster. Violation of this condition is called *contamination* and can dilute the treatment effect estimate. Contamination would occur in this trial if *Wolbachia*-infected mosquitoes migrate into control clusters and/or if participants travel to areas with a different intervention status than their assigned cluster. To protect against mosquito migration, clusters were designed to be separated by a buffer zone of at least 200 m and by natural barriers known to inhibit mosquito movement. *Wolbachia* prevalence and *Aedes aegypti* mosquito abundance will be tracked over time in both intervention and control clusters to assess for contamination. Human movement is not expected to be substantial due to the age (6–11 years) of our participants but will be tracked through a time-use survey administered to at least 20 participants per cluster. A supporting analysis of the primary outcome may explicitly account for measured contamination by tailoring an existing or proposed method to our design [64, 69].

For the primary outcome of the EVITA trial, we chose a composite outcome of total infection by the three major ABV known to have recently circulated in the study population (DENV, ZIKV, and CHIKV) by annual serologic surveillance. The one potential drawback of this approach is that our primary outcome is not a direct measure of disease, and the ultimate goal of this work is to reduce disease burden by ABV. Additionally, there are some limitations in the serologic assessment of ARBV infection, particularly for closely related flaviviruses (FLAV) such as DENV and ZIKV that may circulate in the same population [53, 70, 71]. The potential challenge of determining study endpoints is mitigated by the natural epidemiology at our study site in that very few young children are expected to have already experienced two FLAV infections. Additionally, to meet the primary endpoint, discrimination to the virus species level is not required. Thus, we will be able to discern DENV from ZIKV in the vast majority of participants. For those with clear FLAV infection, but unclear species of infecting virus, the infection still contributes to the primary endpoint as well as multiple secondary endpoints.

There are also several advantages to choosing total infection as the outcome of interest. From a cost-efficiency standpoint, it is more expeditious to conduct annual serosurvey campaigns based at the participating schools, whereas evaluating every acute febrile illness in a pediatric cohort of over 3000 participants is a resource-intensive undertaking. Biologically, disease is necessarily a subset of infections, and it does not seem likely that a partially effective intervention would selectively reduce inapparent ABV infections while case counts remain unaltered. Choosing a composite outcome of DENV, ZIKV, and CHIKV infections maximizes the ability to capture events, which could be particularly important if there were an epidemic year for ZIKV or CHIKV during the study. Finally, the power calculation for the study was determined specifically for DENV because sufficient epidemiologic data was available for that virus. Thus, any infections by ZIKV or CHIKV effectively increase study power, but the study is sufficiently powered without these provided the historic pattern of DENV transmission continues during the study period.

A trial of this type and magnitude creates opportunities to address many questions and hypotheses. We sought to include additional objectives that could be reasonably addressed within the existing trial design as exploratory objectives. Additionally, attention was given to create valuable resources for future investigation, including collection and archiving of entomologic specimens and storage of human biospecimens for future use. A few exploratory objectives merit specific mention. To fully assess the impact of the intervention, direct and indirect evidence of impact will be measured by pulling data generated by activities external to trial operations. For example, we will analyze publicly available regional data on the incidence of dengue and severe dengue for periods before, during, and after trial activity. We will also determine whether ARBV control efforts reduce absenteeism in the study arms by comparing school attendance records. We will assess how well cluster assignment corresponds to a child’s actual location (discussed briefly above) via time-use surveys. An interesting indirect but convenient approach to assessing the impact of vector control efforts involves serologic assays that indicate bite burden. Humans may mount antibody responses to mosquito species-specific salivary proteins, and the presence or level of these antibody responses could correlate to mosquito bite exposure [72, 73].

There are several challenges that could face a clinical trial of this magnitude in school-aged children. These include the current COVID-19 pandemic, low retention rates, low ARBV transmission seasons in BH, and inadequate levels of introgression of *w*Mel-BH in treatment clusters. For example, the COVID-19 pandemic affected trial recruitment since schools were closed to prevent the spread of SARS-CoV-2. To adapt to this situation, recruitment processes were modified to home-based and health center-based encounters. If pandemic-related school closures were to persist throughout the trial follow-up period, this could introduce new challenges for retention. The true event rate (ARBV infection) for the study period could vary considerably from what is projected. The natural transmission pattern for ARBV is seasonal and exhibits high and low transmission seasons that are not precisely predictable. While DENV surveillance data from BH suggest that three consecutive low DENV seasons are highly unlikely, it is not impossible. Finally, the deployment of *w*Mel-BH mosquitoes must result in sufficient introgression to exert the full effect of the intervention. For the establishment of *w*Mel-BH, this is an iterative process that may not proceed uniformly in all clusters due to human population density, the abundance of wild *Aedes aegypti* mosquitoes, and other environmental factors.

## Conclusion

The EVITA Dengue trial is a landmark trial with a school-based cluster-randomized design that was launched in September 2020 in Belo Horizonte with plans for completion in 2023. The trial will determine the efficacy of *w*Mel-BH mosquitoes in preventing transmission of DENV, ZIKV, and CHIKV in the municipality of BH, Brazil. We anticipate that the trial will provide robust evidence needed to guide practice and policy for vector control to combat ABV.

## Trial status

This manuscript reflects protocol version 5, which was IRB approved on September 12, 2021.

Recruitment began on September 8, 2020, and was completed on December 18, 2020.

## Supplementary Information

**Additional file 1.** SPIRIT 2013 Checklist.

**Additional file 2: Figure S1.** Original projected study timeline from Protocol Version 3.

**Additional file 3.** Portuguese and English versions of Consent forms.

**Additional file 4.** Portuguese and English versions of Assent forms.

## Data Availability

The full protocol will be made available within 1 year of the conclusion of data collection. Study details and results will be made publicly available via https://clinicaltrials.gov/ compatible with all regulations and requirements of NIAID.
