# Supplementary figures and images for: EVITA Dengue: a cluster-randomized controlled trial to EValuate the efficacy of Wolbachia-InfecTed Aedes aegypti mosquitoes in reducing the incidence of Arboviral infection in Brazil
Source: Trials. 2022 Mar 2;23:185. doi: 10.1186/s13063-022-05997-4 (PMC8889395; doi:10.1186/s13063-022-05997-4)

**Figure S1.** Original projected study timeline from Protocol Version 3.

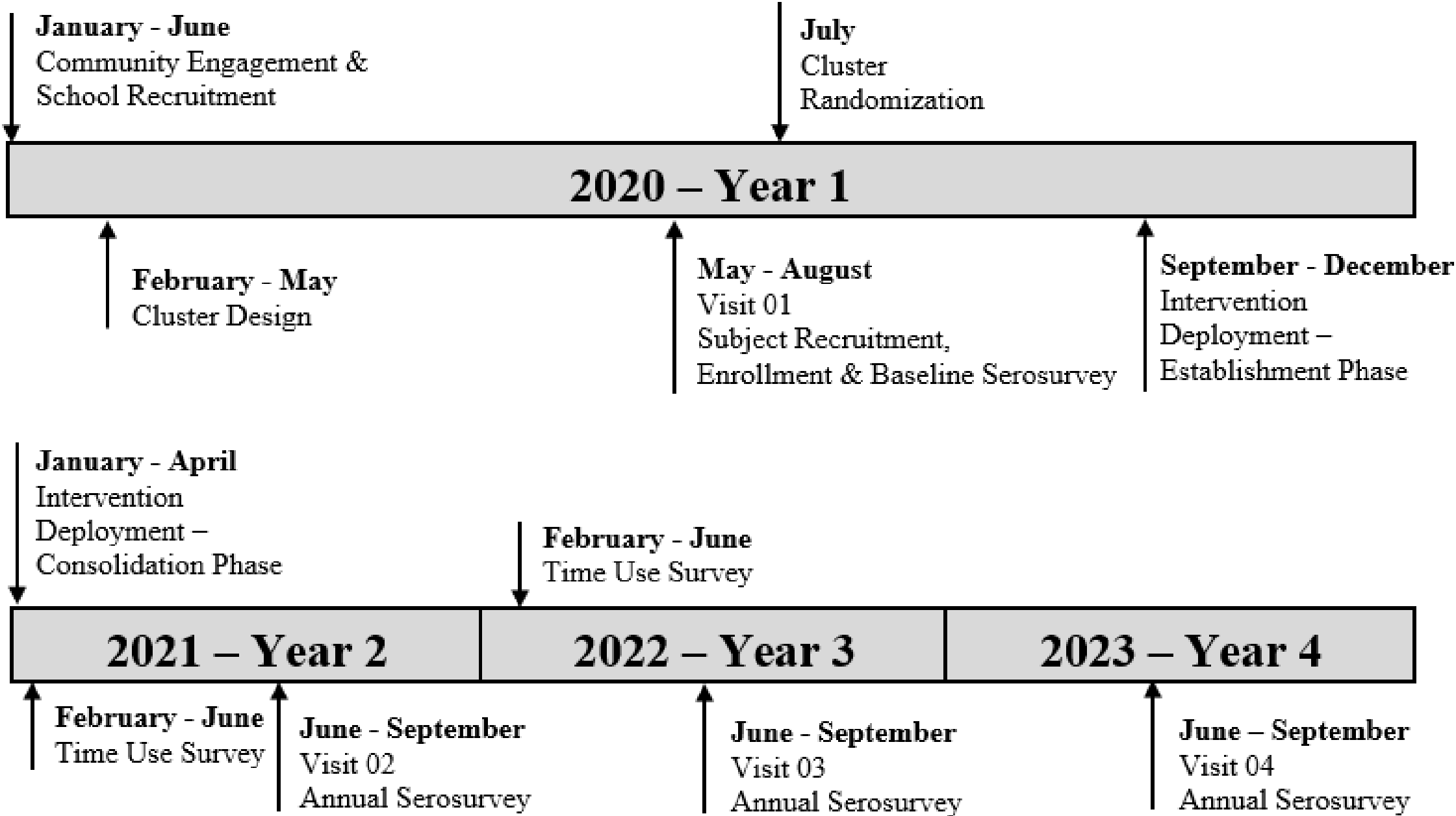

Supplement: Supplementary file 2 — Additional file 2: Figure S1. Original projected study timeline from Protocol Version 3. [file 13063_2022_5997_MOESM2_ESM.zip › Additional file 2/EVITA DENV reResub Original Timeline Figure S1-ADD4.pdf]

## Slide 1
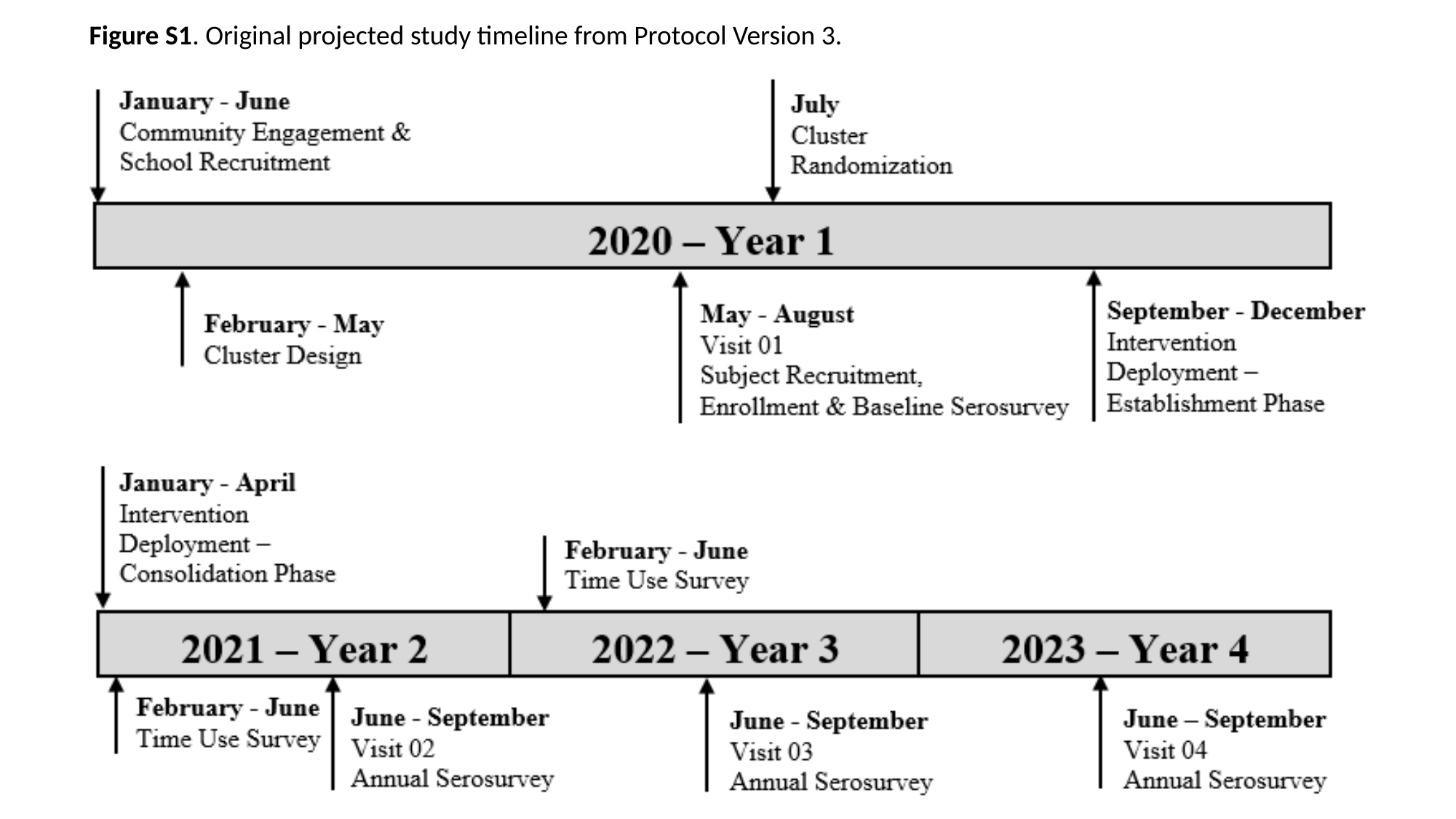

Figure S1. Original projected study timeline from Protocol Version 3.

Supplement: Supplementary file 2 — Additional file 2: Figure S1. Original projected study timeline from Protocol Version 3. [file 13063_2022_5997_MOESM2_ESM.zip › Additional file 2/EVITA DENV reResub Original Timeline Figure S1-ADD4.pptx]
