# Supplementary material for: EVITA Dengue: a cluster-randomized controlled trial to EValuate the efficacy of Wolbachia-InfecTed Aedes aegypti mosquitoes in reducing the incidence of Arboviral infection in Brazil
Source: Trials. 2022 Mar 2;23:185. doi: 10.1186/s13063-022-05997-4 (PMC8889395; doi:10.1186/s13063-022-05997-4)
Supplement: Supplementary file 3 — Additional file 3. Portuguese and English versions of Consent forms. [file 13063_2022_5997_MOESM3_ESM.zip › Additional file 3/TCLE_v4_23out2020_Aprvd 5.11.21-ADD2.pdf]

TERMO DE CONSENTIMENTO LIVRE E ESCLARECIDO (PAIS E/OU RESPONSÁVEIS)

Este termo de consentimento pode conter algumas palavras que não são familiares para você.

Pergunte à equipe do estudo sobre qualquer coisa que você não entenda ou qualquer coisa sobre a qual deseje mais esclarecimentos.

Título do Protocolo: Ensaio randomizado em cluster para avaliação da eficácia de mosquitos *Aedes aegypti* infectados com *Wolbachia* na redução da incidência de infecção por arbovírus no Brasil- Projeto EVITA DENGUE

Patrocinador: Divisão de Microbiologia e Doenças Infecciosas da Universidade de Emory, EUA e o Instituto Nacional de Saúde-NIH, EUA

Apoio: WMP- Programa Mundial de Mosquitos

**Responsável Local**	**Investigador Principal**		**Telefone**
	**Nome**	**Função**	
Universidade Federal de Minas Gerais - UFMG	Dr. Mauro Martins Teixeira	Investigador Principal	(31) 3409-2651<br>(31) 99516-6160

Seu filho(a) e/ou dependente está sendo convidado a participar deste estudo porque tem entre 6 e 11 anos de idade e frequenta uma escola em Belo Horizonte que foi escolhida para fazer parte deste Projeto.

Este documento explica como será o estudo e qual a participação do seu filho(a) e/ou dependente nele. Por favor, leia-o atentamente. Leve o tempo necessário para decidir se realmente deseja que seu filho(a) e/ou dependente participe do estudo. Você pode levar este documento para casa e conversar com seus familiares antes de tomar a decisão. Após ser esclarecido(a) sobre as informações a seguir e, caso aceite que seu filho(a) e/ou dependente faça parte do estudo, rubrique cada página e assine ao final deste documento, que está em duas vias. Uma delas é sua e a outra do pesquisador responsável.

Não assine esse termo caso você não tenha entendido completamente os procedimentos e riscos do estudo, ou caso não esteja completamente satisfeito com as explicações dadas a você.

Introdução

As arboviroses são um grave problema de saúde pública sendo causa de internação de milhares de pessoas no Brasil e no mundo, podendo levar à morte. As arboviroses mais comuns são as causadas pelos vírus da Dengue, Zika e Chikungunya. Essas doenças podem ser transmitidas através da picada do mosquito *Aedes aegypti*. Vários métodos de controle tem sido utilizados pela prefeitura de Belo Horizonte a fim de combater o mosquito transmissor da doença e diminuir a ocorrência dessas doenças. No entanto, apesar desse esforço, continuamos a ter epidemias sazonais de arboviroses como a dengue na nossa cidade.

Objetivo deste estudo

Rubrica do pesquisador:_________________Rubrica do Responsável pelo Participante: ____

O objetivo deste estudo é verificar se a liberação de mosquitos *Aedes aegypti* especialmente modificados no meio ambiente reduz o número de pessoas infectadas pela Dengue, Zika e/ou Chikungunya.

Esses mosquitos *Aedes aegypti* modificados possuem um microrganismo chamado *Wolbachia* que não é prejudicial às pessoas. Pesquisas indicam que a presença da *Wolbachia* nos mosquitos faz com eles não consigam transmitir os arbovírus aos humanos. Ao serem soltos no meio ambiente, eles acasalam com mosquitos selvagens e produzem mosquitos que também possuem a *Wolbachia*.

Como será o estudo?

O seu filho(a) e/ou dependente está sendo convidado a participar do estudo que será realizado em diversas regiões de Belo Horizonte. Essas regiões foram divididas em várias zonas (conhecidas como clusters) com base em escolas da rede municipal de ensino. Em metade desses clusters, haverá a liberação no ambiente dos mosquitos com *Wolbachia* e na outra metade não haverá a liberação.

Selecionamos 58 escolas municipais para participar do estudo e em cada uma convidaremos 60 crianças.

Descrição dos procedimentos

O estado de saúde do seu filho(a) e/ou dependente será acompanhado durante todo o estudo e quatro amostras do sangue dele serão coletadas e analisadas para verificar se ele teve Dengue, Zika e/ou Chikungunya, mesmo que não tenha apresentado sintomas. Isto vai acontecer uma vez por ano, durante quatro anos.

A primeira visita é chamada de visita de triagem ou visita 1. Nessa visita, você também responderá a perguntas sobre a saúde geral do seu filho(a) e/ou dependente. Como os mosquitos só serão liberados em certas áreas, precisamos saber onde seu filho(a) e/ou dependente mora para que possamos rastrear o número de infecções em áreas com ou sem mosquitos liberados.

Após a inclusão no estudo precisaremos coletar uma amostra de sangue. Nos próximos 3 anos, iremos realizar uma visita de acompanhamento anual (visitas 2, 3 e 4), na qual será colhida uma amostra de sangue em cada visita. Todas as visitas de acompanhamento, provavelmente, serão realizadas na escola onde ele(a) estuda. Cada amostra de sangue será de 10ml (uma colher de sobremesa). Os pesquisadores testarão o sangue do seu filho(a) e/ou dependente para detectar a presença de infecções transmitidas pelo mosquito e a exposição a picadas de mosquito. Essas informações ajudarão os pesquisadores a determinar se a liberação dos mosquitos modificados pode prevenir essas infecções.

Quem pode participar do estudo?

Crianças entre 6 e 11 anos, regularmente matriculadas em uma escola selecionada da rede publica municipal de Belo Horizonte, que residam e permaneçam por pelo menos 5 dias da semana na área correspondente a sua escola.

Quais os possíveis riscos ao seu filho(a) e/ou dependente?

No local da coleta de amostra de sangue: a retirada de amostras de sangue pode causar dor, vermelhidão ou manchas roxas. Para minimizar todos esses riscos, as coletas serão realizadas por profissionais treinados, com experiência e o material sempre será descartável e esterilizado.

Rubrica do pesquisador:_________________Rubrica do Responsável pelo Participante: _____

O que acontecerá com a amostra de sangue do seu filho(a) e/ou dependente?

Serão coletadas amostras de sangue do seu filho(a) e/ou dependente em todas as visitas do estudo com o objetivo de testá-lo para doenças transmitidas por mosquitos *Aedes aegypti* como a Dengue, Zika e Chikungunya.

Essas amostras serão processadas e uma alíquota (parte) será enviada para os testes sorológicos no laboratório credenciado pelo estudo, localizado em São José do Rio Preto- SP e para as outras análises no laboratório nos Estados Unidos da América (EUA).

Para preservar o anonimato, no lugar do nome do seu filho(a) e/ou dependente constará na amostra apenas o número da sua identificação no estudo. Se você tiver alguma preocupação ou dúvidas sobre o envio das amostras ou quiser retirar sua permissão para o armazenamento delas, você pode entrar em contato com o pesquisador na escola do seu filho(a) e/ou dependente ou através do telefone disponibilizado neste termo. A qualquer momento as amostras podem ser destruídas, se for da sua vontade. Ressaltamos que nenhum dos testes previstos envolve o material genético do seu filho(a) e/ou dependente, uma vez que os testes são apenas para procurar anticorpos contra Dengue, Zika e Chikungunya.

Armazenamento de amostras biológicas do estudo em Biorrepositório

Uma parte das amostras de sangue do seu filho(a) e/ou dependente, coletadas durante o estudo, será armazenada no Brasil em um Biorrepositório na UFMG (coleção de material biológico armazenado nos laboratórios do estudo) e a outra parte será enviada para os EUA (Biorrepositório DMID- EUA), para realizar testes previstos no estudo e serem usadas em estudos futuros.

O envio de amostras para os EUA para realizar testes previstos no estudo se faz necessário porque nossos parceiros no exterior possuem a capacidade e a competência necessárias para realizar os testes laboratoriais propostos neste estudo. Todos os exames laboratoriais feitos serão realizados com a participação de pesquisadores do grupo de pesquisa desse estudo.

As amostras biológicas (sangue) coletadas durante este estudo serão armazenadas de acordo com as normas do Conselho Nacional de Saúde que regulam o armazenamento de material biológico humano ou uso de material armazenado em pesquisas. As amostras podem ser armazenadas por até 10 anos (Resolução nº441/2011), podendo ser utilizadas em estudos futuros, desde que estes projetos sejam aprovados pelo Comitê de Ética em Pesquisa, e você assine um novo termo de consentimento desta nova pesquisa.

O endereço do Biorrepositório no Brasil é:

Dr. Mauro Martins Teixeira - (31) 3409-2651 ou (31) 99516 6160
Universidade Federal de Minas Gerais (UFMG)
End: Instituto de Ciências Biológicas da UFMG (ICB/UFMG), Bloco G3, Sala 101. Av. Pres. Antônio Carlos, 6627, CEP: 31270-901, Belo Horizonte/MG

=

O Biorrepositório nos EUA, ficará aos cuidados do patrocinador do estudo DMID. O endereço nos EUA é:

Rubrica do pesquisador:_________________Rubrica do Responsável pelo Participante: ____

**DMID - Clinical Materials Services (CMS) Fisher
BioServices
20439 Seneca Meadows Parkway - Germantown, MD 20876
Phone: 240-477-1350 - Fax: 240-477-1360
E-mail: DMID.CMS@ThermoFisher.com**

As pesquisas futuras que utilizem o material biológico do seu filho(a) e/ou dependente poderão ser realizadas para investigar fatores relacionados à predisposição, tratamento e prevenção de doenças infecciosas, bem como contribuir para o aprimoramento de novos testes diagnósticos dessas doenças.

Sobre o uso do material biológico armazenado (sangue), assinale uma das opções abaixo:

( ) Concordo que o material biológico do meu filho(a) e/ou dependente seja utilizado somente para esta pesquisa.

( ) Concordo que o material biológico do meu filho(a) e/ou dependente possa ser utilizado nesta pesquisa e em pesquisas futuras (Biorrepositório), mas serei comunicado pelo pesquisador novamente e assinarei outro termo de consentimento livre e esclarecido que explique para que será utilizado o material.

Caso você não concorde com o armazenamento das amostras, estas serão descartadas imediatamente ao final do estudo.

Durante os exames previstos na pesquisa pode sobrar material residual (sangue) que poderia ser jogado fora, entretanto, queremos aproveitar esse material, deixando-os armazenados para pesquisas futuras.

( ) Sim, concordo com o armazenamento das amostras residuais do meu filho(a) e/ou dependente no Biorrepositório do Instituto Nacional de Saúde (NIH), Divisão de Microbiologia e Doenças Infecciosas (DMID).

( ) Não concordo com o armazenamento das amostras residuais do meu filho(a) e/ou dependente no Biorrepositório do Instituto Nacional de Saúde (NIH), Divisão de Microbiologia e Doenças Infecciosas (DMID). Portanto todas as amostras residuais deverão ser destruídas ao final da pesquisa.

É importante ressaltar que:

- Você é livre para ceder ou não o material biológico do seu filho(a) e/ou dependente e sua decisão não lhe causará quaisquer prejuízos.
- Você é livre para desautorizar o uso das amostras e dados relacionados ao material biológico do seu filho(a) e/ou dependente em qualquer fase da pesquisa, sem prejuízo ou penalização alguma.
- As informações pessoais serão mantidas em sigilo e privacidade, uma vez que o material biológico do seu filho(a) e/ou dependente será codificado para que nenhuma informação permita identificá-lo.
- Você será avisado sobre a necessidade de descarte do material armazenado no Biorrepositório. O descarte poderá acontecer se a amostra não atender a critérios mínimos de qualidade para a

Rubrica do pesquisador:_________________Rubrica do Responsável pelo Participante: ____

MOD003-STUDY00000239
IRB Approved
5/11/2021

pesquisa, se houver dificuldades de arquivo (espaço físico, por exemplo) ou se o Biorrepositório deixar de existir.

- A legislação brasileira proíbe o patenteamento e a utilização comercial de material biológico humano armazenado em Biorrepositórios. A amostra do seu filho(a) e/ou dependente somente será usada para fins de pesquisa científica.

A realização de outras pesquisas futuras utilizando o material depositado no Biorrepositório do DMID necessita obrigatoriamente de aprovação do Comitê de Ética em Pesquisa institucional e quando for o caso da Comissão Nacional de Ética em Pesquisa (CONEP).

Desistência do Estudo

Se seu filho(a) e/ou dependente se tornar um participante, ele poderá retirar-se deste estudo a qualquer momento. Para retirá-lo, você pode ligar para um membro da equipe de pesquisa e dizer-lhes que seu filho não quer mais participar. Isso cancelará quaisquer compromissos futuros para o estudo. Os pesquisadores também podem retirá-lo da pesquisa, se necessário. A retirada do estudo não implicará qualquer penalidade ou perda de benefícios a que você tenha direito.

Se houver a retirada do estudo, nenhuma informação de saúde nova será reunida após essa data. As informações que já foram coletadas podem ser usadas até o final do estudo de pesquisa, conforme necessário para garantir a integridade e/ou supervisão do estudo. Além disso, os dados que foram coletados até o momento da retirada do seu filho serão mantidos e analisados permitindo uma avaliação completa e abrangente do estudo.

Em caso de desconforto ou reações adversas

Se o seu filho(a) e/ou dependente tiver algum desconforto, como resultado da participação neste estudo e necessitar de ajuda, entre em contato com a equipe do estudo para que eles saibam o que está acontecendo. Eles podem ajudá-lo a obter os cuidados necessários. As informações para contato podem ser encontradas ao final deste termo.

O seu filho(a) e/ou dependente tem direito a assistência integral e gratuita pelo tempo que for necessário devido aos danos decorrentes da participação na pesquisa. De acordo com as normas brasileiras ele tem direito a requerer indenização em caso de danos decorrentes da pesquisa.

Quais as alternativas para o seu filho(a) e/ou dependente sobre participar neste estudo de pesquisa?

Você pode escolher que seu filho(a) e/ou dependente não participe do estudo. Os direitos não serão afetados ou alterados se você optar por ele(a) não participar do estudo, ou mesmo deixá-lo, caso já tenha iniciado.

Confidencialidade

Qualquer informação identificável obtida em conexão com este estudo será mantida na clínica do estudo em um local seguro com acesso restrito. Eles somente serão divulgados com sua permissão ou conforme exigido pelas leis estaduais ou federais brasileiras. Apenas a equipe do estudo, o patrocinador do estudo,

Rubrica do pesquisador:_________________Rubrica do Responsável pelo Participante: ____

os comitês de ética que aprovaram o estudo e as agências reguladoras do Brasil e dos Estados Unidos podem ter acesso às suas informações, mas não terão permissão para identificá-lo como participante do estudo. Todas essas pessoas manterão suas informações privadas. Não forneceremos nenhuma informação que o identifique a ninguém que não esteja trabalhando no estudo.

Para ajudar a proteger a sua confidencialidade, usaremos códigos de identificação nos dados da pesquisa que serão armazenados nas salas do estudo em arquivos trancados Apenas a equipe de pesquisa e aqueles que supervisionam a pesquisa têm acesso aos dados da pesquisa. Os dados eletrônicos serão armazenados em computadores e sites protegidos por senha. Para este estudo, cada amostra de sangue será etiquetada apenas com um código de barras e um número de rastreamento exclusivo para proteger sua confidencialidade. O pessoal envolvido no armazenamento das amostras e do laboratório central de testes não saberá sua identidade ou o código de identificação (ID) atribuído a você para o estudo. Se escrevermos um relatório ou artigo sobre este estudo ou compartilharmos os dados do estudo com outras pessoas, faremos isso de forma que você não possa ser identificado diretamente, a menos que seu consentimento específico para esta atividade seja obtido.

Dúvidas

Utilizamos alguns termos técnicos neste formulário de consentimento. Por favor, sinta-se à vontade para perguntar sobre qualquer coisa que não entenda antes de tomar uma decisão.

Lembramos que não é prevista nenhuma ajuda financeira pela sua participação, pois a aceitação é voluntária e não está sujeita a nenhum tipo de compensação monetária. Este estudo não prevê nenhum procedimento, exceto a coleta de sangue anual.

Se você tiver alguma dúvida quanto aos direitos do seu filho(a) e/ou dependente ou quiser esclarecer dúvidas a respeito dessa pesquisa, você pode contatar os pesquisadores responsáveis por telefone (inclusive ligações a cobrar) ou pelo e-mail: **Evitadengue@gmail.com**

Contato da Equipe do Estudo

Se você tiver qualquer dúvida e quiser falar com a equipe do estudo é só entrar em contato:

Universidade Federal de Minas Gerais (UFMG)
Médico Responsável: Dr. Mauro Martins Teixeira
Telefones: (31) 99516-6160 e (31) 3409-2651
Endereço: Instituto de Ciências Biológicas da UFMG (ICB/UFMG), Bloco G3, Sala 101 Av. Pres. Antônio Carlos, 6627, CEP: 31270-901, Belo Horizonte/MG

Em caso de dúvidas relacionadas aos aspectos éticos deste estudo, você poderá consultar: Comitê de Ética em pesquisa da Universidade Federal de Minas Gerais – UFMG (COEP-UFMG)

Av. Presidente Antônio Carlos, 6627 – Campus Pampulha- Unidade Administrativa II – 2º Andar - Sala: 2005, telefone: (31) 3409-4592 - Belo Horizonte, Minas Gerais, CEP 31270- 901- E-mail: coep@prpq.ufmg.br

Pode consultar também:

Rubrica do pesquisador:_________________Rubrica do Responsável pelo Participante: ____

Comissão Nacional de Ética em Pesquisa – CONEP

SRTV 701, Via W 5 Norte, lote D - Edifício PO 700, 3º andar – telefones: (61) 3315-5893 ou 5883 ou 5886 ou 5891- Asa Norte - Brasília-DF- CEP: 70719-040, e-mail: conep.cep@saude.gov.br

CONSENTIMENTO DA PARTICIPAÇÃO DA PESSOA COMO SUJEITO

Eu,____, CPF_____ _______________abaixo assinado, concordo com a participação do meu filho(a) e/ou dependente___ ______________________nascido(a) em/______________________________/_______, no estudo "Ensaio randomizado em cluster para avaliação da eficácia de mosquitos Aedes aegypti infectados com Wolbachia na redução da incidência de infecção por arbovírus no Brasil - Projeto EVITA DENGUE", como voluntário(a). Fui devidamenteinformado(a) e esclarecido(a) pelo pesquisador(a) _______________sobre a pesquisa, os procedimentos nela envolvidos, assim como os possíveis riscos e benefícios decorrentes da participação do meu filho(a) e/ou dependente. Foi-me garantido o sigilo das informações e que posso retirar meu consentimento a qualquer momento, sem nenhuma penalidade. Assinando esse termo, eu consinto em fornecer as amostras biológicas do meu filho(a) e/ou dependente para o estudo e no seu armazenamento na instituição patrocinadora para fins de pesquisa. Li e entendi as informações acima. Tive a oportunidade de fazer perguntas e esclarecer todas as minhas dúvidas. Este formulário está sendo assinado voluntariamente por mim. Concordo com a participação do meu filho(a) e/ou dependente até que eu decida o contrário. Receberei uma via assinada desse consentimento.

____

Nome do responsável legal pelo participante (como escrito no documento de identidade)

____

Assinatura do responsável pelo participante

Data______/______/______

____,

Testemunha (para casos de participantes analfabetos, semianalfabetos ou portadores de deficiência auditiva ou visual): Nome conforme escrito no documento de identidade

____

Assinatura da testemunha

Data______/______/______

____

Rubrica do pesquisador:_________________Rubrica do Responsável pelo Participante: _____

MOD003-STUDY00000239
IRB Approved
5/11/2021

Nome do profissional que obteve consentimento

____

Assinatura do profissional que obteve o consentimento

Data______/______/______

Nome do participante: ____
Escola do participante:____
ID do participante: ____
Data de nascimento:_____/_____/______.

Rubrica do pesquisador:_________________Rubrica do Responsável pelo Participante: ____
