# Supplementary material for: EVITA Dengue: a cluster-randomized controlled trial to EValuate the efficacy of Wolbachia-InfecTed Aedes aegypti mosquitoes in reducing the incidence of Arboviral infection in Brazil
Source: Trials. 2022 Mar 2;23:185. doi: 10.1186/s13063-022-05997-4 (PMC8889395; doi:10.1186/s13063-022-05997-4)
Supplement: Supplementary file 3 — Additional file 3. Portuguese and English versions of Consent forms. [file 13063_2022_5997_MOESM3_ESM.zip › Additional file 3/TCLE_v4_23out2020_English_Aprvd 5.11.21-ADD2.pdf]

FREE AND CLARIFIED CONSENT FORM (PARENTS AND / OR GUARDIANS)

This consent form may contain some words that are unfamiliar to you.

Ask the study team about anything you don't understand or anything you want further clarification on.

Protocol Title: A cluster randomized trial to evaluate the efficacy of Wolbachia-infected Aedes aegypti to reduce the incidence of arboviral infection in Brazil

Sponsor: Division of Microbiology and Infectious Diseases, Emory University, USA and the National Institute of Health-NIH, USA

Support: WMP- World Mosquito Program

Local Site Responsible	Principal Investigator		Phone
	Name	Occupation	
Universidade Federal de Minas Gerais - UFMG	Dr. Mauro Martins Teixeira	Principal Investigator	(31) 3409-2651<br>99516 6160

Your child and / or dependent is being invited to participate in this study because he is between 6 and 11 years old and attends a school in Belo Horizonte that was chosen to be part of this Project.

This document explains what the study will be like and what your child's participation and / or dependent will participate in. Please read it carefully. Take the time to decide whether you really want your child and / or dependent to participate in the study. You can take this document home and talk to your family members before making a decision. After being clarified on the following information and, if you accept that your child and / or dependent is part of the study, initial each page and sign at the end of this document, which is in two copies. One is yours and the other is by the responsible researcher.

Do not sign this term if you have not fully understood the study's procedures and risks, or if you are not completely satisfied with the explanations given to you.

Introduction

Arboviruses are a serious public health problem that causes thousands of people to be hospitalized in Brazil and worldwide and can lead to death. The most common arboviruses are those caused by the Dengue, Zika and Chikungunya viruses. These diseases can be transmitted through the bite of the *Aedes aegypti* mosquito. Various control methods have been used by the city of Belo Horizonte in order to combat the mosquito that transmits the disease and reduce the occurrence of these diseases. However, despite this effort, we continue to experience seasonal epidemics of arboviruses such as dengue in our city.

Researcher Initials:______________________________Legal Guardian Initials: __________________________

Purpose of the Study

The objective of this study is to verify if the release of specially modified Aedes aegypti mosquitoes in the environment reduces the number of people infected with Dengue, Zika and / or Chikungunya.

These modified *Aedes aegypti* mosquitoes have a microorganism called *Wolbachia* inside of them that is not harmful to people. Research indicates that the presence of *Wolbachia* in mosquitoes makes them unable to transmit arboviruses to humans. When released into the environment, they mate with wild mosquitoes and produce mosquitoes that also have *Wolbachia* in them.

How will the study work?

Your child and / or dependent is being invited to participate in the study that will be carried out in several regions of Belo Horizonte. These regions were divided into several zones (known as clusters) based on schools in the municipal school system. In half of these clusters, *Wolbachia* mosquitoes will be released into the environment and in the other half of clusters, there will be no releases.

We selected 58 municipal schools to participate in the study. We will enroll 60 children from each school.

Description of Procedures

Your child and / or dependent's health status will be monitored throughout the study. Four blood samples will be collected and analyzed to see if he/she has had Dengue, Zika and / or Chikungunya, even if he/she has no symptoms. This will happen once a year for four years.

The first visit is called a screening visit or visit 1. During this visit, you will also answer questions about the general health of your child and / or dependent. Since the mosquitoes will only be released in certain areas, we need to know where your child and / or dependent lives so that we can track the number of infections in areas with or without released mosquitoes.

After inclusion in the study, we will need to collect a blood sample. Over the next 3 years, we will carry out annual follow-up visits (visits 2, 3 and 4), in which a blood sample was taken at each visit. All follow-up visits will probably take place at the school where he/she studies. Each blood sample will be 10ml (the size of a dessert spoon). Researchers will test your child and / or dependent's blood to detect the presence of mosquito-borne infections and exposure to mosquito bites. This information will help researchers determine whether the release of the modified mosquitoes can prevent these infections.

Researcher Initials:______________________________Legal Guardian Initials: __________________________

Who can participate in the study?

Children between 6 and 11 years old, regularly enrolled in a school selected from the municipal public network of Belo Horizonte, who reside and stay for at least 5 days a week in the area corresponding to their school.

What are the possible risks to your child and / or dependent?

At the blood sample collection site: taking blood samples can cause pain, redness or bruising. To minimize all these risks, the collections will be carried out by trained, experienced professionals and the material will always be disposable and sterilized.

What will happen to your child's and / or dependent's blood sample?

Blood samples will be collected from your child and / or dependent on all study visits in order to test them for diseases transmitted by *Aedes aegypti* mosquitoes such as Dengue, Zika and Chikungunya.

These samples will be processed and an aliquot (part) will be sent for serological tests in the laboratory accredited by the study, located in São José do Rio Preto-SP and for the other analyses in the laboratories in the United States of America (USA).

In order to preserve anonymity, instead of your child and / or dependent's name, only a study identification number will appear in the sample. If you have any concerns or questions about sending the samples or want to withdraw your permission to store them, you can contact the researcher at your child and / or dependent's school or through the phone provided in this term. At any time, the samples can be destroyed, if you wish. We emphasize that none of the tests provided for involves the genetic material of your child and / or dependent, since the tests are only to look for antibodies against Dengue, Zika and Chikungunya.

Storage of Biological Samples from the Study in a Biorepository

A portion of your child and / or dependent's blood samples collected during the study will be stored in Brazil in a Biorepository at UFMG (collection of material stored in the study laboratories) and the other part will be sent to the USA (DMID Biorepository), to carry out study tests and to be used in future research studies.

Sending samples to the USA to carry out the tests provided for in the study is necessary because our partners abroad have the necessary capacity and competence to carry out the laboratory tests proposed in this study. All laboratory tests performed will be performed with the participation of researchers from the research group of this study.

The biological samples (blood) collected during this study will be stored in accordance with the rules of the National Health Council that regulate the storage of human biological material or use of material stored in research. The samples

can be stored for up to 10 years (Resolution nº 441/2011), and can be used in future studies, as long as these projects are approved by the Research Ethics Committee, and you sign a new consent form for this new research.

The address of the Biorepository in Brazil is:

Dr. Mauro Martins Teixeira - (31) 3409-2651 ou (31) 99516 6160
Universidade Federal de Minas Gerais (UFMG)
End: Instituto de Ciências Biológicas da UFMG (ICB/UFMG), Bloco G3, Sala 101. Av. Pres. Antônio Carlos, 6627, CEP: 31270-901, Belo Horizonte/MG

The Biorepository in the USA will be in the care of the DMID study sponsor. The U.S. address is:

DMID – Clinical Materials Services (CMS)
Fisher BioServices
20439 Seneca Meadows Parkway - Germantown, MD 20876
Phone: 240-477-1350 - Fax: 240-477-1360
E-mail: DMID.CMS@ThermoFisher.com

Future research using the biological material of your child and / or dependent may be carried out to investigate factors related to the predisposition, treatment and prevention of infectious diseases, as well as to contribute to the improvement of new diagnostic tests for these diseases.

Regarding the use of stored biological material (blood), check one of the options below:

( ) I agree that the biological material of my child and / or dependent is used only for this research.

( ) I agree that my child and / or dependent's biological material can be used in this research and in future research (Biorepository), but I will be communicated by researcher again and I will sign another informed consent form that explains what the material will be used for.

If you do not agree with the storage of the samples, they will be discarded immediately at the end of the study.

During the exams provided for in the research there may be residual material (blood) that could be thrown away, however, we want to take advantage of this material, leaving it stored for future research.

( ) Yes, I agree with the storage of the residual samples of my child and / or dependent in the Biorepository of the National Institute of Health (NIH), Division of Microbiology and Infectious Diseases (DMID).

( ) I do not agree with the storage of the residual samples of my child and / or dependent in the Biorepository of the National Institute of Health (NIH), Division of Microbiology and Infectious Diseases (DMID). Therefore, all residual samples must be destroyed at the end of the research.

It is important to note that:

Researcher Initials:______________________________Legal Guardian Initials: __________________________

- You are free to transfer or not your child and / or dependent's biological material and your decision will not cause any harm to you.
- You are free to disallow the use of samples and data related to your child and / or dependent's biological material at any stage of the research, without prejudice or penalty.
- Personal information will be kept confidential and private, since your child and / or dependent's biological material will be encoded so that no information can identify you.
- You will be advised of the need to dispose of the material stored in the Biorepository. Discarding may happen if the sample does not meet minimum quality criteria for the research, if there are file difficulties (physical space, for example) or if the Biorepository ceases to exist.
- Brazilian law prohibits the patenting and commercial use of human biological material stored in biorepositories. Your child and / or dependent's sample will only be used for scientific research purposes.

Other future research using the material deposited in the DMID Biorepository requires the approval of the Institutional Research Ethics Committee and when it is the case of the National Research Ethics Committee (CONEP).

Withdrawal from the Study

If your child and / or dependent becomes a participant, they can withdraw from this study at any time. To get it out, you can call a member of the research team and tell them that your child no longer wants to participate. This will cancel any future appointments for the study. Researchers can also withdraw your child and / or dependent from the survey, if necessary. Withdrawing from the study will not entail any penalty or loss of benefits to which you are entitled.

If you withdraw your child and / or dependent from the study, no new health information will be collected after that date. Information that has already been collected can be used until the end of the research study, as needed to ensure the integrity and / or oversight of the study. In addition, the data that was collected up to the time of your child and / or dependent's withdrawal will be maintained and analyzed allowing for a complete and comprehensive assessment of the study.

In case of discomfort or adverse reactions

If your child and / or dependent has any discomfort as a result of participating in this study and needs help, contact the study team to let them know what is going on. They can help you to get the necessary care. Contact information can be found at the end of this form.

Researcher Initials:______________________________ Legal Guardian Initials: __________________________

Your child and / or dependent is entitled to full and free assistance for as long as necessary due to the damage resulting from participating in the research. According to Brazilian rules, he/she is entitled to claim compensation in case of damages resulting from the research.

What are the alternatives for your child and / or dependent on participating in this research study?

You can choose to not have your child and / or dependent participate in the study. Their rights will not be affected or changed if you choose not to participate in the study, or even leave it, if you have already started.

Confidentiality

Any identifiable information obtained in connection with this study will be kept at the study clinic in a secure place with restricted access. It will only be disclosed with your permission or as required by Brazilian state or federal law. Only the study staff, the sponsor of the study, the ethics committees that have approved the study, and the Brazilian and United States regulatory agencies, may have access to your information but will not be allowed to identify you as a study participant. All of these people will keep your information private. We will not give any information that identifies you to anyone who is not working on the study.

To help protect your confidentiality, we will use identification codes in the research data that will be stored in the study rooms in locked files. Only the research team and those who supervise the research have access to the research data. Electronic data will be stored on password protected computers and websites. For this study, each blood sample will be labeled with only a barcode and a unique tracking number to protect its confidentiality. The personnel involved in storing the samples and the central testing laboratory will not know your identity or the identification code (ID) assigned to you for the study. If we write a report or article about this study or share the study data with others, we will do so that you cannot be identified directly, unless your specific consent to this activity is obtained.

Questions

We use some technical terms in this consent form. Please feel free to ask about anything you don't understand before making a decision.

We remind you that no financial aid is provided for your participation, as acceptance is voluntary and is not subject to any type of monetary compensation. This study does not foresee any procedure other than the annual blood collection.

If you have any questions regarding the rights of your child and / or dependent or want to ask questions about this research, you can contact the researchers responsible by phone (including collect calls) or by email: Evitadengue@gmail.com.

Study Team Contact

MOD003-STUDY00000239
IRB Approved
5/11/2021

If you have any questions and want to speak with the study team, just get in touch:

Dr. Mauro Martins Teixeira- (31) - 3409-2651 ou (31) 99516 6160
Universidade Federal de Minas Gerais (UFMG)
End: Instituto de Ciências Biológicas da UFMG (ICB/UFMG), Bloco G3, Sala 101. Av. Pres. Antônio Carlos, 6627, CEP: 31270-901, Belo Horizonte/MG

If you have any questions related to the ethical aspects of this study, you can contact:

Research Ethics Committee of the Federal University of Minas Gerais - UFMG (COEP-UFMG) Av. Presidente Antônio Carlos, 6627 - Campus Pampulha- Administrative Unit II - 2nd Floor - Room: 2005, telephone: (31) 3409-4592 - Belo Horizonte, Minas Gerais, CEP 31270- 901- E-mail: coep@prpq.ufmg.br

You can also contact:

National Research Ethics Commission - CONEP

SRTV 701, Via W 5 Norte, lot D - PO 700 Building, 3rd floor - phones: (61) 3315-5893 or 5883 or 5886 or 5891- Asa Norte - Brasília-DF- CEP: 70719-040, e-mail: conep.cep@saude.gov.br

CONSENT TO CHILD AND / OR DEPNENDENT'S PARTICIPATION AS A SUBJECT

I,____,CPF______

the undersigned, agree to my child's participation,_________________born on ________/_______/______, in the study "Randomized cluster trial to assess the effectiveness of Aedes aegypti mosquitoes infected with *Wolbachia* in reducing the incidence of arbovirus infection in Brazil - Project EVITA DENGUE", as a volunteer. I was duly informed and clarified by the researcher ________________________about the research, the procedures involved in it, as well as the possible risks and benefits resulting from the participation of my child and / or dependent. I was guaranteed the confidentiality of the information and that I can withdraw my consent at any time, without any penalty. By signing this term, I consent to provide the biological samples of my child and / or dependent for the study and its storage at the sponsoring institution for research purposes. I read and understood the information above. I had the opportunity to ask questions and clarify all my questions. This form is being voluntarily signed by me. I agree with the participation of my child and / or dependent until I decide otherwise. I will receive a signed copy of that consent.

Researcher Initials:____________________________Legal Guardian Initials: ________________________

MOD003-STUDY00000239
IRB Approved
5/11/2021

____

Name of the legal guardian for the participant (as written on the identity document)

____

Signature of the person responsible for the participant

Date_______/_______/_______

____,

Witness (for cases of illiterate, semi-illiterate or hearing or visually impaired participants): Name as written on the identity document

____

Witness signature

Date_______/_______/_______

____

Name of the professional who obtained consent

____

Signature of the professional who obtained the consent

Date_______/_______/_______

Name of Participant: ____
School of Participant:____
Participant Study ID: ____
Date of Birth: ______/_________/_______.

Researcher Initials:_____________________________Legal Guardian Initials: __________________________
