# Supplementary material for: EVITA Dengue: a cluster-randomized controlled trial to EValuate the efficacy of Wolbachia-InfecTed Aedes aegypti mosquitoes in reducing the incidence of Arboviral infection in Brazil
Source: Trials. 2022 Mar 2;23:185. doi: 10.1186/s13063-022-05997-4 (PMC8889395; doi:10.1186/s13063-022-05997-4)
Supplement: Supplementary file 4 — Additional file 4. Portuguese and English versions of Assent forms. [file 13063_2022_5997_MOESM4_ESM.zip › Additional file 4/TALE_v4_23out2020_Aprvd 5.11.21-ADD3.pdf]

TERMO DE ASSENTIMENTO LIVRE E ESCLARECIDO

(Crianças de 7 a 11 anos)

Título do Protocolo: Ensaio randomizado em cluster para avaliação da eficácia de mosquitos Aedes aegypti infectados com Wolbachia na redução da incidência de infecção por arbovírus no Brasil- EVITA DENGUE

Patrocinador: Divisão de Microbiologia e Doenças Infecciosas da Universidade de Emory, EUA e o Instituto Nacional de Saúde-NIH, EUA

Apoio: WMP- Programa Mundial de Mosquitos

Responsável Local	Investigador Principal		Telefone
	Nome	Função	
Universidade Federal de Minas Gerais - UFMG	Dr. Mauro Martins Teixeira	Investigador Principal	(31) 3409-2651<br>99516 6160

Você está sendo convidado para participar deste estudo porque você tem entre 7 e 11 anos de idade e é aluno de uma escola municipal que foi uma das escolas de Belo Horizonte escolhidas para fazer parte deste Projeto. É importante que você entenda o que é este projeto antes de decidir se você vai participar ou não dele. Nós já conversamos com os seus pais e/ou responsáveis e eles sabem que estamos pedindo sua ajuda. Se alguma coisa que eu disser aqui, você não entender, pode me pedir que eu explico na mesma hora.

Você não precisa falar AGORA se quer participar ou não, pois você poderá levar uma via deste documento para casa e conversar com seus familiares antes de se decidir. Se você não quiser participar desse projeto não tem nenhum problema, você não é obrigado. Mesmo se os seus pais e/ou responsáveis concordarem é você quem vai decidir se quer ou não. Se você concordar em participar e depois mudar de ideia também não tem problema, você poderá desistir quando quiser.

O QUE É O PROJETO?

Quero lhe fazer uma pergunta. Você sabe o que é um vírus? Vírus são micróbios pequenininhos que podem causar doenças se estiverem no nosso corpo. Existem três doenças chamadas dengue, chikungunya e zika, você já ouviu falar delas? Pois então, os vírus que causam estas doenças vivem nos mosquitos que a gente conhece como “mosquito da dengue”. O nome verdadeiro desse mosquito é *Aedes aegypti* e quem tomar uma picada desse mosquito pode contrair uma dessas três doenças. Você sabia que existem vários cientistas e médicos que querem diminuir o número de pessoas que ficam doentes por causa das picadas desses mosquitos? E nós estamos aqui justamente para isso.

Este projeto é uma PESQUISA CIENTÍFICA, que está acontecendo aqui em Belo Horizonte. Nesta pesquisa queremos saber como podemos diminuir as doenças causadas pelos vírus que o “mosquito da dengue” transmite.

Então, nós pensamos em um plano que pode ajudar e estamos convidando várias crianças para fazer parte desse plano. Os cientistas criaram mosquitos especiais que mesmo se eles picarem as pessoas, eles não conseguem transmitir essas doenças, e que talvez, se nós soltarmos esses mosquitos especiais em Belo Horizonte nós teremos menos pessoas com doenças causadas pelos mosquitos. Você entendeu? E

Rubrica do pesquisador:_________________Rubrica do Participante: ___________________

MOD003-STUDY00000239
IRB Approved
5/11/2021

o que você acha de nos ajudar com esse plano?

COMO SERÁ?

Esse projeto vai durar quatro anos e a sua participação é muito importante. Para saber se você já teve alguma dessas doenças, os cientistas precisam tirar um pouquinho de sangue do seu braço. Isto acontecerá quatro vezes, a primeira vez será ainda este ano, depois, apenas uma vez por ano, nos próximos três anos. Não se preocupe porque vai demorar bastante entre uma vez e outra. Você percebeu que o seu sangue é o mais importante para que esse projeto funcione? Pois é assim que os cientistas saberão se está dando certo nossa pesquisa com os mosquitos especiais que soltamos.

NINGUÉM VAI SABER! (É UM SERGREDO NOSSO!)

Nós não vamos contar para ninguém que você está participando desta pesquisa e nem vamos colocar seu nome em lugar nenhum. Somente você, seus pais e/ou responsáveis e nós cientistas que vamos saber da sua participação.

VOCÊ PARTICIPA SE QUISER!

Mesmo se o seus pais e/ou responsável autorizar, você não é obrigado a participar. E você pode sair do projeto a qualquer momento. Nós não vamos ficar chateados com você se você mudar de ideia

O QUE VOCÊ PRECISA FAZER?

Se quiser participar você tem que:

Primeiro: Assinar esse documento, ele é chamado de Termo de Assentimento-TALE e comprova que você entendeu tudo que nós conversamos agora. Se os seus pais (ou responsáveis) concordarem com sua participação neste projeto e você quiser participar e assinar este documento, nós vamos conversar novamente para que você nos responda outras perguntas que vão ajudar muito os cientistas na pesquisa. Isto é importante para termos certeza que você pode fazer parte do estudo. Você receberá uma via desse documento e a outra ficará guardada com a gente.

Segundo: precisaremos de tirar a primeira amostra do seu sangue**.** Sabemos que pode incomodar e até doer um pouquinho, pode até ficar um pouco vermelho ou roxo, mas nossa equipe é bem treinada e fará da melhor forma para que você fique tranquilo e se sinta bem.

Pedimos que você nos conte sempre que ficar doente. Seus pais irão levá-lo ao seu médico, mas nós também precisamos saber. Toda semana uma pessoa da nossa pesquisa visitará a sua escola. Você pode contar para ela ou pedir a seus pais para nos ligar.

CONTATO DA EQUIPE DO ESTUDO

Se você tiver qualquer dúvida e quiser falar com a equipe do estudo é só pedir para seus pais ou responsáveis entrarem em contato

Rubrica do pesquisador:_________________Rubrica do Participante: ___________________

MOD003-STUDY00000239
IRB Approved
5/11/2021

Nome do Centro de Pesquisa Clínica: Universidade Federal de Minas Gerais (UFMG)
Médico Responsável: Dr. Mauro Martins Teixeira
Telefones: 31- 99516 6160 e 3409 2651

Endereço: Instituto de Ciências Biológicas da UFMG (ICB/UFMG),Bloco G3, Sala 101– Av. Pres. Antônio Carlos, 6627,CEP: 31270-901, Belo Horizonte/MG

Em caso de dúvidas relacionadas aos aspectos éticos deste estudo, seus pais ou responsáveis poderão consultar:

Comitê de Ética em pesquisa da Universidade Federal de Minas Gerais – UFMG (COEP-UFMG)

Av. Presidente Antônio Carlos, 6627 – Campus Pampulha- Unidade Administrativa II – 2º Andar - Sala: 2005, telefone: (31) 3409-4592 - Belo Horizonte, Minas Gerais, CEP 31270- 901- E-mail: coep@prpq.ufmg.br

Pode consultar também:

Comissão Nacional de Ética em Pesquisa – CONEP

SRTV 701, Via W 5 Norte, lote D - Edifício PO 700, 3º andar – telefones: (61) 3315-5893 ou 5883 ou 5886 ou 5891- Asa Norte - Brasília-DF- CEP: 70719-040, e-mail: conep.cep@saude.gov.br

Nome do participante: ____
Escola do participante:____
ID do participante: ____
Data de nascimento: _____/_____/______.

Caso você concorde em participar da pesquisa coloque seu nome aqui:

____,
(Nome do participante)

Data______/______/______.

____

Nome do profissional que obteve consentimento

____

Assinatura do profissional que obteve o consentimento

Data______/______/______

Rubrica do pesquisador:_________________Rubrica do Participante: ___________________
