# Supplementary material for: EVITA Dengue: a cluster-randomized controlled trial to EValuate the efficacy of Wolbachia-InfecTed Aedes aegypti mosquitoes in reducing the incidence of Arboviral infection in Brazil
Source: Trials. 2022 Mar 2;23:185. doi: 10.1186/s13063-022-05997-4 (PMC8889395; doi:10.1186/s13063-022-05997-4)
Supplement: Supplementary file 4 — Additional file 4. Portuguese and English versions of Assent forms. [file 13063_2022_5997_MOESM4_ESM.zip › Additional file 4/TALE_v4_23out2020_English_Aprvd 5.11.21-ADD3.pdf]

MOD003-STUDY00000239
IRB Approved
5/11/2021

FREE AND CLARIFIED CONSENT FORM
(Children from 7 to 11 years old)

Protocol Title: A cluster randomized trial to evaluate the efficacy of Wolbachia-infected Aedes aegypti to reduce the incidence of arboviral infection in Brazil

Sponsor: Division of Microbiology and Infectious Diseases, Emory University, USA and the National Institute of Health-NIH, USA

Support: WMP- World Mosquito Program

Local Site Responsible	Principal Investigator		Phone
	Name	Occupation	
Universidade Federal de Minas Gerais - UFMG	Dr. Mauro Martins Teixeira	Principal Investigator	(31) 3409-2651<br>99516 6160

You are being invited to participate in this study because you are between 7 and 11 years old and you are a student at a municipal school that was one of the schools in Belo Horizonte chosen to be part of this project. It is important that you understand what this project is about before deciding whether or not to participate in it. We have already talked to your parents and / or guardians and they know that we are asking for your help. If there is anything I say here that you don't understand, you can ask me to explain it right away. You do not need to speak NOW if you want to participate or not, as you will be able to take a copy of this document home and talk to your family members before deciding. If you do not want to participate in this project, that is not a problem, you are not obliged to take part. Even if your parents and / or guardians agree, it is up to you to decide whether or not you want to participate. If you agree to participate and then change your mind, that's fine too, you can leave the study whenever you want.

WHAT IS THE PROJECT ABOUT?

I want to ask you a question. Do you know what a virus is? Viruses are tiny microbes that can cause disease if they are in our body. There are three diseases called dengue, chikungunya and zika. Have you heard of them? The viruses that cause these diseases live in mosquitoes that we know as the “dengue mosquito”. The real name of this mosquito is *Aedes aegypti* and whoever gets bitten by one of these mosquitoes can contract one of these three diseases. Did you know that there are several scientists and doctors who want to decrease the number of people who get sick because of these mosquito bites? And we are here for just that.

Researcher Initials:______________________________Participant Initials: ______________________________

This project is SCIENTIFIC RESEARCH that is happening here in Belo Horizonte. In this research we want to know how we can reduce the diseases caused by the viruses that the “dengue mosquito” transmits. So, we have come up with a plan that can help, and we are inviting several children to be part of that plan. Scientists have created special mosquitoes that, even if they bite people, they cannot transmit these diseases. Perhaps, if we release these special mosquitoes in Belo Horizonte, we will have fewer people with diseases caused by mosquitoes. Does that make sense? And what do you think about helping us with this plan?

WHAT WILL HAPPEN ON THE STUDY?

This project will last four years, and your participation is very important. To find out if you have ever had any of these diseases, scientists need to draw a little blood from your arm. This will happen four times, the first time this year, then only once a year for the next three years. Don't worry, there will be long periods of time between drawing your blood. Did you realize that your blood is the most important part for this project to work? This is how scientists will know if our research with the special mosquitoes we have released is working.

NO ONE WILL KNOW! (IT'S OUR SECRET!)

We will not tell anyone that you are participating in this survey, nor will we put your name anywhere. Only you, your parents and / or guardians and we scientists will know about your participation.

YOU PARTICIPATE IF YOU WANT!

Even if your parents and / or guardian authorizes it, you are not required to participate. And you can leave the project at any time. We won't be upset with you if you change your mind.

WHAT DO YOU NEED TO DO?

If you want to participate you have to:

First: Sign this document. It is called the Term of Assent (TALE) and proves that you understood everything we talked about now. If your parents (or guardians) agree to participate in this project and you want to participate and sign this document, we will talk again so that you can answer other questions that will help scientists a lot in this research. This is important to make sure you can be part of the study. You will receive one copy of this document and the other will be kept with us.

Second, we will need to take the first sample of your blood. We know that it can be annoying and even hurt a little. It may even be a little red or purple, but our team is well trained and will do the best we can to make you feel calm and good.

We ask that you tell us whenever you get sick. Your parents will take you to your doctor, but we

MOD003-STUDY00000239
IRB Approved
5/11/2021

also need to know. Every week a person from our study will visit your school. You can tell her or ask her parents to call us.

STUDY TEAM CONTACT

If you have any questions and would like to speak with the study team, just ask your parents or guardians to contact us.

**Name of Clinical Research Center: Universidade Federal de Minas Gerais (UFMG)**
**Doctor Responsible: Dr. Mauro Martins Teixeira**
**Phone Number: 31- 99516 6160 e 3409 2651**
**Address: Instituto de Ciências Biológicas da UFMG (ICB/UFMG),Bloco G3, Sala 101– Av. Pres. Antônio Carlos, 6627,CEP: 31270-901, Belo Horizonte/MG**

If you have questions related to the ethical aspects of this study, your parents or guardians can contact:

Research Ethics Committeê at the Federal University of Minas Gerais - UFMG (COEP-UFMG) Av. Presidente Antônio Carlos, 6627 – Campus Pampulha- Unidade Administrativa II – 2º Andar - Sala: 2005, telefone: (31) 3409-4592 - Belo Horizonte, Minas Gerais, CEP 31270- 901- E-mail: coep@prpq.ufmg.br

You can also contact:

Comissão Nacional de Ética em Pesquisa – CONEP

SRTV 701, Via W 5 Norte, lote D - Edifício PO 700, 3º andar – telefones: (61) 3315-5893 ou 5883 ou 5886 ou 5891- Asa Norte - Brasília-DF- CEP: 70719-040, e-mail: conep.cep@saude.gov.br

Name of Participant: ____ Sc

If you agree to participate in the survey put your name here:

____,

(Participant Name).

Date _____/______/______.

____

Name of the professional who obtained consent

____

Signature of the professional who obtained the consent

Date_______/_______/_______

Researcher Initials:______________________________Participant Initials: ______________________________

MOD003-STUDY00000239
IRB Approved
5/11/2021

Researcher Initials:______________________________Participant Initials: ______________________________
